# An updated insight on testicular hemodynamics: Environmental, physiological, and technical perspectives in farm and companion animals

**DOI:** 10.1007/s11259-022-10022-9

**Published:** 2022-11-18

**Authors:** Haney Samir, Mohamed I. ElSayed, Faten Radwan, Mohamed Hedia, Hanan Hendawy, Amin Omar Hendawy, Mohamed Elbadawy, Gen Watanabe

**Affiliations:** 1 Department of Theriogenology, Faculty of Veterinary Medicine, Cairo University, Giza, 12211 Egypt; 2 Laboratory of Veterinary Physiology, Department of Veterinary Medicine, Faculty of Agriculture, Tokyo University of Agriculture and Technology, 3-5-8 Saiwai-Cho, Fuchu, Tokyo 183-8509 Japan; 3 Veterinarian graduated from the Faculty of Veterinary Medicine, Benha University, Moshtohor, Toukh, 13736 Elqaliobiya Egypt; 4 Department of Veterinary Surgery, Faculty of Veterinary Medicine, Suez Canal University, Ismailia, 41522 Egypt; 5 Laboratory of Veterinary Surgery, Tokyo University of Agriculture and Technology, Tokyo, 183-8509 Japan; 6 Department of Animal and Poultry Production, Faculty of Agriculture, Damanhour University, Damanhour, 22516 Egypt; 7 Department of Pharmacology, Faculty of Veterinary Medicine, Benha University, Moshtohor, Toukh, 13736 Elqaliobiya Egypt

**Keywords:** Color Doppler ultrasonography, Farm and companion animals, Spermatogenesis, Testicular blood flow, Thermal stress

## Abstract

In all organs, control of blood flow is important but might be particularly critical for testicular functions. This is because of the very low oxygen concentration and high metabolic rate of the seminiferous tubules, the physiological temperature of the testis, and its location outside the abdominal cavity. Many factors affect the characteristics of TBF in farm and companion animals, such as environment (thermal and seasonal effects) and physiological (species, breeds, age, body weight, and sexual maturity). Thermal environment stress has detrimental effects on spermatogenesis and consequently has more serious impacts on both human and animal fertility. Numerous studies have been performed to assess TBF in different animal species including bulls, rams, bucks, alpacas, stallions, and dogs with varied results. Hence, assessment of TBF by Doppler ultrasonography is of great importance to estimate the effect of high environment temperature on testicular functions. Also, differences observed in the TBF may result from different technical aspects such as the identification of the segment of the testicular artery to be examined. In the current review, we focused on the imperative roles of TBF in various animal species. Besides, we discussed in detail various factors that could affect TBF. These factors can significantly modify the TBF and thus should be considered when establishing reference values in farm animals for better clinical diagnosis. The information provided in this review is valuable for researchers and veterinarians to help them a better understanding of testicular hemodynamics for the proper evaluation of breeding soundness examination in males.

**Supplementary Information:**

The online version contains supplementary material available at 10.1007/s11259-022-10022-9.

## Importance of blood flow to the testis

In the practice of animal reproduction, especially ruminants, a limited number of males with peak reproductive efficiency are selected to impregnate many females in the same breeding system (Barth and Waldner 2002; Camela et al. 2019). Therefore, a proper assessment of the reproductive efficiency of the male is very important. There are different methods to evaluate the breeding potential of male farm and companion animals. A comprehensive breeding soundness evaluation (BSE) of a male has an established list of procedures for each species. The evaluation of semen quality is the most important step in the routine examination of breeding soundness (Ax et al. 2000). The soundness of the male reproductive system can be determined by various methods, including ultrasound examinations of the reproductive tract, especially the testis (Ortiz-Rodriguez et al. 2017). Rarely, endocrine evaluation of circulating hormones such as follicle-stimulating hormone (FSH), luteinizing hormone (LH), testosterone, and estradiol may help determine the breeding capacity of some animal species, such as stallions (Douglas and Umphenour 1992).

Testicular blood flow (TBF) is the key pathway for the transport of nutrients, oxygen, regulatory hormones, and other secretory products to and from the testicular tissues. In addition to its imperative roles in the transport of oxygen and nutrient supply, blood flow also plays pivotal roles in testicular thermoregulation (Junior et al. 2018). Blood flow is defined as the total amount of blood that moves past a certain point. When blood flows through a vessel, it is affected by two factors (pressure and resistance) and expressed as flow = pressure/resistance (Pinggera et al. 2008). The testicular artery is the target of TBF assessment because the testis receives its blood supply exclusively through this vessel. Because of its coiled appearance, the testicular artery is unusually long (up to 220 cm in bulls, 145 to 215 cm in buffalo, 180–225 cm and up to 400 cm in rams, and 137–170 cm in equine) (Harrison 1949; Setchell et al. 1966; Elayat et al. 2014; Khalil 2014).

Testicular blood flow is distinguished by a high vascular resistance; at the convoluted portion of the testicular artery (supratesticular artery; STA) compared with other parts of this vessel, which results in a lowering of the intratesticular capillary pressure as compared with other organs (Sweeney et al. 1991; Bergh and Damber 1993; Trautwein et al. 2019). This low-pressure results in an environment of low oxygen tension inside the seminiferous tubules (Ortiz-Rodriguez et al. 2017).

Spermatogenesis is adapted to a semihypoxic environment, which provides sperm cells with the advantage of avoiding damage caused by oxygen-free radicals (Max 1992; Setchell et al. 1994; Aitken 1999). Although low oxygen tension is very beneficial to spermatogenesis (Bergh and Damber 1993), the testis is very liable to suffer from ischemic damage when blood perfusion is decreased due to vascular restriction (Kay et al. 1992). Therefore, the early identification of changes in the TBF is required for a correct diagnosis of various testicular disorders and prompt implementation of appropriate treatment (Ortega-Ferrusola et al. 2014). The experimental restriction of TBF leads to reduction in the testicular size and great impairment in spermatogenesis in bulls (Kay et al. 1992). In rams, various degrees of testicular ischemia induced focal morphological changes in the testes (Markey et al. 1995). Pathological disorders affecting the testis's vasculature, such as varicocele or spermatic cord torsion, greatly reduced TBF and compromised testicular function in stallions (Pozor 2007). Importantly, a mild decrease in TBF has detrimental effects on early-stage spermatogenesis (Nolte et al. 1995; Bergh et al. 2001). In rats (Hsu et al. 1994), experimental varicocele induced significant reductions in TBF and defective adenine nucleotide concentrations, and energy charge as the result of inadequate nutrient supply from the blood circulation. These findings could result in defective energy metabolism at the mitochondrial level (due to decreased synthesis of adenosine 5'-triphosphate; ATP), damage and ischemia of testicular tissues, and in turn impairment of spermatogenesis.

## Anatomical consideration of the testicular artery

The testicular artery is derived from the aorta. It runs along the inguinal canal and forms numerous irregular loops termed the funicular part of the testicular artery or supratesticular artery (STA), which form a cone-like structure. Here the great coiling of the vessels disperses the heat, resulting in a reduction in the working temperature of the testis (Kastelic et al. 1997). The degree of testicular artery coiling varies by animal species. Bulls (up to 130 loops), rams (80 loops), and bucks (50 loops) all have a lot of coiling in their testicular artery. In other animal species such as stallions, camels, and dogs, the coiling is comparatively smaller (less than 25 loops), whereas humans have no coiling in the testicular artery (Harrison and Weiner 1949; Setchell 1978; Gouletsou 2017). Variations among animal species in the degree of convolutions of the testicular artery might be attributed to the differences in abdominal–testicular temperature gradients (Harrison and Weiner 1949).

In ruminants, the testicular artery branches into a testicular and epididymal branch after leaving the pampiniform plexus (Kastelic et al. 1997; Gouletsou 2017). The testicular branch runs along the posterior margin of the testis and is named the marginal portion; the marginal testicular artery (MTA). The MTA is very long concerning the position and the size of the testis in ruminants. So, it has a degree of tortuosity, especially in rams and bucks compared to the bulls. At the ventral pole of the testis (on the level of the junction between the proximal and distal parts of the epididymal border of the testis), it forms a sigmoid curve and then soon branches into lateral and medial testicular arteries (Elayat et al. 2014). The lateral and medial testicular arteries wheel the tail, then split into 7–9 smaller branches that travel to both surfaces of the testis in a dorsolateral and dorsomedial direction. The “tunica arteriosa testis” is formed by these branches running in a wavy pattern and adhering closely to one another. The parenchymal branches are 20 delicate, slender vessels that emerge from the tunica arteriosa testis and run the length of the testicular surface. Then, they run through the testicular parenchyma in a radiating fashion toward the mediastinum testis as centripetal parenchymal branches (Elayat et al. 2014). At the mediastinum, they overturn themselves and return in an opposite direction and parallel to the centripetal branches as centrifugal parenchymal branches, finally joining the mediastinum to the subcapsular space (Polguj et al. 2015; Gouletsou 2017).

There are some morphological variations between ruminants' testicular arteries and those of other animal species. The testicular artery in stallions, for example, travels in a wavy pattern along the epididymal edge (known as the marginal portion of the testicular artery), along the caudal testicular pole, and along the free edge of the testis as one to three branches (Collin 1973; Pozor 2007). The MTA encircles the testicular circumference completely along both borders (Elayat et al. 2014). On the ventral aspect of the testis, small arterial branches run on the lateral and medial testicular surfaces toward the epididymal edge, until they penetrate the tunica albuginea toward the testicular parenchyma as centripetal arteries (Jantosovicová and Jantosovic 1983). In dogs, the convoluted part (named the STA) of the testicular artery is cranial to the testis, while the MTA runs in a straight plane to the caudal pole of the testis, and gives off many branches on either side, from which terminal vessels pass into the testicular parenchyma and directed towards the center of the testis (Harrison and Weiner 1949). The proximal portion of the STA (cranial segment of the STA) is loosely convoluted, while the distal portion (looping segment of the STA) is of high convolutions as the artery approached the cranial pole of the testis (Harrison and Weiner 1949).

## TBF evaluation methods

Vascular patterns are investigated using various methods such as arteriography and microarteriography after the injection of radiopaque media into the testicular artery, with subsequent observation of the relation between the testicular veins and the artery in histological transverse sections (Barclay 1947; Jantosovicová and Jantosovic 1983). Although these methods were used a long time ago, they are still very useful modalities for the evaluation of the vasculature of various organs and often provide good anatomical features of the organ’s blood supply (branches and other parts) (Khadamy et al. 2018). These methods were not designed to provide velocimetric measures of blood flow. Whereas arteriography and microarteriography provide only anatomical details of various blood vessels, laser Doppler flowmetry can be used to measure microvascular TBF in various animal species (Widmark et al. 1986; Gonzalvo et al. 1993).

Advances in Doppler applications have enabled researchers and veterinarians to properly assess the structural and functional aspects of the testis (Ortiz-Rodriguez et al. 2017; Fávaro et al. 2020; El-Sherbiny et al. 2022a). Doppler ultrasonography depends on the phenomena of the Doppler effect, which was first described in 1842 by Christian Johann Doppler. It is a natural phenomenon characterized by apparent changes in the frequency of the sound wave when the source of the sound wave moves toward or away from the receptor. Differences between the generated and received frequencies are referred to as the “Doppler shift,” which is proportional to the velocity of the movement. Likewise, in ultrasound waves, the difference between the frequency of the received and transmitted echoes by the transducer is known as Doppler frequency (Zagzebski 2005; Herzog and Bollwein 2007). The frequency differences occur via the movement of blood cells and allow for the detection and measurement of blood flow (Ginther 2007; Viana et al. 2013).

There is no difference between the transmitted and received frequencies when the receiver target (in this case, red blood cells) is stationary or traveling parallel to the wave source (transducer), and the colorful Doppler signals are not observed. The returned frequency is greater than the transmitted frequency while blood flow is traveling toward the transducer, resulting in a positive Doppler effect. A negative Doppler signal is generated when the returning frequency is lower than the transmitted frequency, or when the red blood cells move away from the transducer (Zagzebski 2005). The greatest frequency shift occurs when the transmitted ultrasound beam is parallel to the blood flow. However, to enable the detection of the blood vessel, there must be a small angle of insonation (≤ 60◦) between the direction of the ultrasound waves and the direction of blood flow (Pozor and McDonnell 2004; Junior et al. 2020; Samir et al. 2021).

There are color Doppler and spectral Doppler modalities or displays. Color Doppler includes directional Doppler and power Doppler, while spectral Doppler includes continuous-wave (CW) Doppler and pulse-wave (PW) Doppler. Color and power Doppler serve a different purpose than using CW or PW Doppler, for example, visualization of vessels vs measuring blood flow velocities. The spectral Doppler modes (PWD, CWD) examine the velocity and direction of blood through a selected small zone, called sample volume, whose velocities are of interest to be examined. Consequently, the examination of this sample volume allows the quantitative analysis of the blood flow characteristics (if compared with color Doppler) in a specific area of the artery (Boon 2011a, b).

The theory of pulsed-wave Doppler imaging is similar to that of B-mode imaging, in which sound is emitted in short bursts and received by the same crystal during the time interval between pulses (only one crystal is used for transmitting and receiving sound). The echoes returning from a vessel would arrive after a certain period if the sample volume in the vessel is set at a specific depth. This time interval (known as range gating) corresponds to the vessel's depth and enables blood flow in a particular vessel to be determined. The exact position of a flow pattern can be calculated in this manner. The pulse repetition frequency must be twice the maximum frequency of the returned echoes to accurately calculate blood-flow velocity (known as the Nyquist limit). When the Nyquist limit is surpassed, an anomaly called aliasing occurs, making correct flow velocity analysis impossible. This can happen if the pulse repetition frequency is too low, if there is high-velocity flow, such as in aortic or pulmonic stenosis, or if the sampling depth is too great. In these situations, it would be appropriate to switch to continuous-wave Doppler ultrasound (Boon 2011a, b).

Continuous-wave Doppler, on the other hand, sends and receives sound in real-time. The transducer is made up of two crystals, one that transmits sound and the other that absorbs it. Sound waves are therefore received continuously, and so continuous-wave Doppler (CW Doppler) can measure very high velocities but is unable to discriminate the depth of the signal. Every moving target in the path of the sound beam will cause a signal and will be measured. Using pulsed-wave Doppler, the origin of the measured velocities must be determined (spectral or color-coded). Animals with heart failure associated with elevated flow rates, such as aortic or pulmonic stenosis, ventricular septal abnormalities, or mitral and tricuspid insufficiency, use CW Doppler to measure flow velocities (Boon 2011a, b).

The Doppler shift in the spectral flow Doppler appears as a chart representing the speed by time (Viana et al. 2013). However, to distinguish between an artery and a vein in this mode, the blood flow within an artery will typically have a spectral graph of waveforms that correspond to the arterial pulsation of the cardiac cycle (systole and diastole). In the veins, the flow of blood will not have a pulsed waveform; almost constant (Herzog and Bollwein 2007).

It is important to point out that measuring accurate velocities is angle-dependent and requires a straight vessel, but calculating Doppler indices, especially resistive index (RI) is not angle-dependent and does not require a straight fragment of the vessel. However, in some tissues of the genital system, such as the ovarian structures (follicles and corpus luteum) and intratesticular parenchyma, the evaluation of blood perfusion may be particularly difficult because of the tortuous orientations and small diameters of the arterioles and venules detectable by ultrasound. In these cases, color Doppler is used, and the cross-section images of the central area or of the areas with the greatest Doppler signal are recorded and later measured using image analysis software to estimate the percentage of color Doppler signals (Pugliesi et al. 2014, 2019; Bollwein et al. 2016; Samir and Kandiel 2019; Samir et al. 2019; Hedia and El-Belely. 2021). Therefore, the application of color flow Doppler provides an overview of the blood flow but gives no detailed information regarding the blood flow velocity parameters of the studied tissue (Viana et al. 2013; Pugliesi et al. 2014, 2019; El-Sherbiny et al. 2022b). Indeed, the STA has similar limitations, due to the tortuosity and the inability to clearly define the angle of insonation. Thereby, depending on Doppler indices (resistive index: RI and pulsatility index: PI) is recommended for a valid assessment of TBF (Ginther and Utt 2004).

## Spectral Doppler parameters for TBF assessment

Spectral Doppler ultrasonography is very important because it translates a detailed analysis of blood flow waveforms and consequently determines blood flow velocity (peak systolic velocity [PSV], end-diastolic velocity [EDV], mean velocity [MV], time average maximum velocity [TAMAX], total arterial blood flow [TABF], and total arterial blood flow rate [TABFR]) and Doppler indices (RI and pulsatility index [PI]) in a particular vessel (Viana et al. 2013). Blood flow in the testicular vessel could be evaluated using the Doppler indices (RI and PI). The resistance of vascular perfusion caused by the microvascular bed distal to the measurement site is referred to as RI, whereas the pulsatility of the waveform is referred to as PI. Increased RI and PI values stipulate decreased distal tissue perfusion. Doppler indices are computed from the values for PSV, EDV, and TAMAX (Ginther and Utt 2004) using these formulas: RI = [(PSV­EDV) ÷ PSV]; PI = [(PSV­EDV) ÷ TAMAX]. Doppler indices are very useful especially when estimating the Doppler angle is difficult (e.g. tortuous vessels). Therefore, these indices are not angle-dependent and are considered good indicators of the downstream flow condition (Blanco et al. 2008; Serin et al. 2010). The blood flow of tissue downstream is negatively associated with RI and PI values in equine (Ginther and Utt 2004). Considering the nonchange in the diameter of the examined vessel, as the values of RI and PI decrease, blood flow resistance reduces, and blood perfusion to the organ improves (Dickey 1997; Bollwein et al. 2016). In general, blood flow pulsatility is higher in larger vessels as compared with smaller vessels (Hassan et al. 2022). In small vessels, Doppler indices are mainly used to determine blood flow semiquantitatively, because it is often nearly impossible to accurately measure the diameter of small vessels (Blanco et al. 2008).

## Factors influencing characterizations of TBF in various animal species

**Fig. 1:**
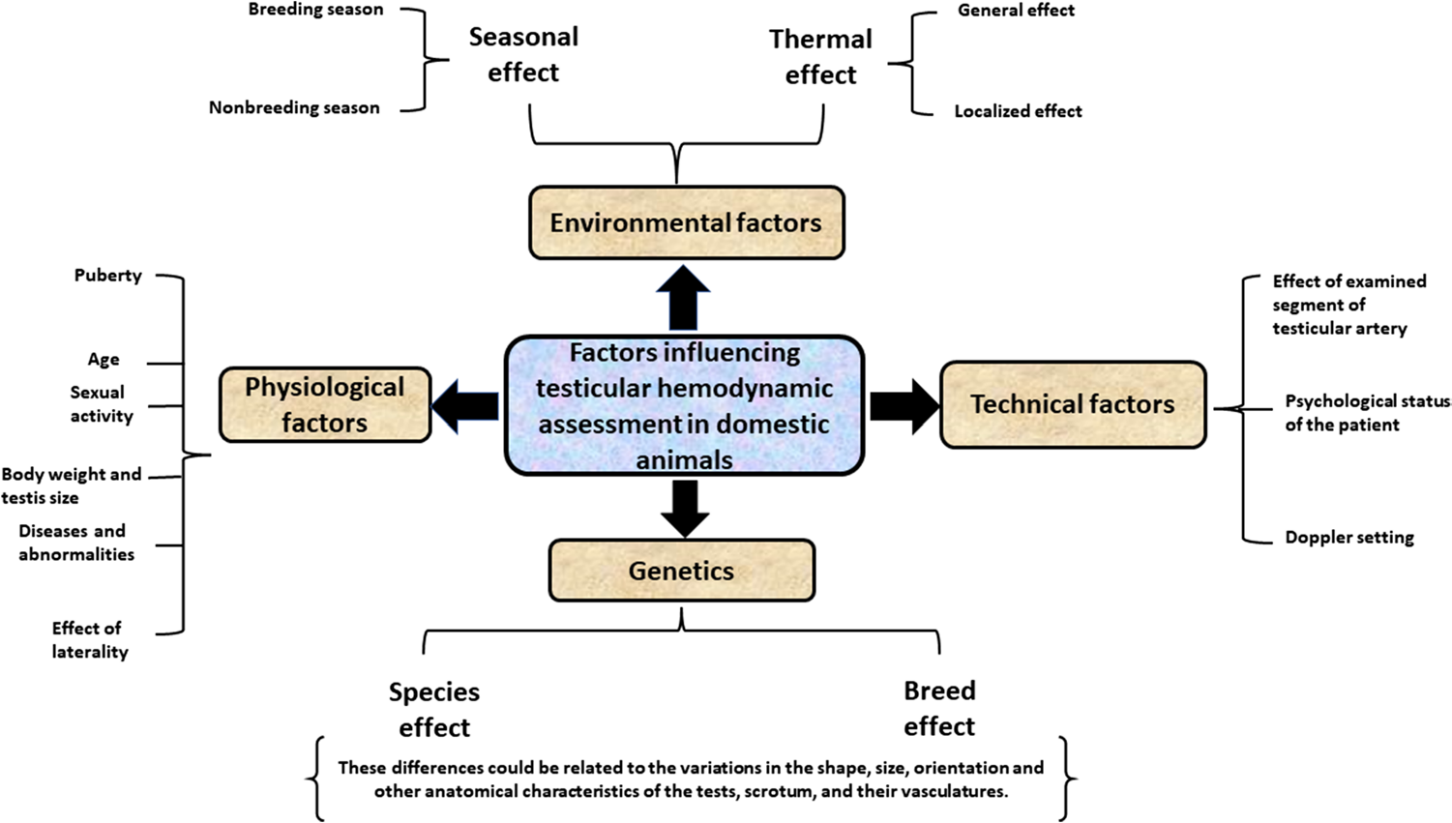
An illustrated diagram of different factors that could influence the testicular blood flow assessment in domestic animals in light of previous bibliographies

To reach a proper diagnosis and maximize the benefits of color Doppler imaging of TBF, it is better to fully understand all factors that might affect testicular blood perfusion in domestic animals (Fig. 1). Many factors affect the characteristics of TBF in farm and companion animals, such as the thermal factor in dogs (Henning et al. 2014) and bulls (Junior et al. 2020), nutritional, anatomical, and genetic (species and breeds) factors in dogs (Souza et al. 2014) and bulls (Junior et al. 2018), and seasonal factors in rams (Ntemka et al. 2018; Hedia et al. 2019) and bucks (Strina et al. 2016; Samir et al. 2018). Likewise, differences in TBF could be observed based on the size or body weight of the animal as well as pubertal differences in dogs (Souza et al. 2014; de Souza et al. 2015a) and rams (Camela et al. 2017, 2019). Other factors can influence TBF, including the age effect in stallions (Pozor and McDonnell 2004) and rams (Ntemka et al. 2018), and sexual activity, ambient temperature, and pathological conditions in stallions (Pozor and McDonnell 2004; Ortiz-Rodriguez et al. 2017).

In this regard. It would be important to highlight the difference between factors that affects blood flow to the testis and those that affect the measurements of the total blood flow by ultrasonographic techniques. For more instances, differences in the results of testicular hemodynamics within the same breed or even within individuals may result from different segments of the testicular artery to be examined and the technique of evaluation in stallions (Pozor and McDonnell 2004) dogs (Gumbsch et al. 2002; Carrillo et al. 2012; de Souza et al. 2014; Trautwein et al. 2019), rams (Hedia et al. 2020a), and bucks (Samir et al. 2020a). The latter could not be necessarily implicated in a real modification of the blood flow to the testis, but it should be addressed for correct diagnosis.

Collectively, these factors can significantly modify the TBF and thus should be considered when setting up reference measures in various animal species such as stallions (Ortega-Ferrusola et al. 2014) for better clinical diagnosis.

Appended, we highlighted the most important factors that could affect TBF in domestic animals. Roughly, four main factors could induce alterations in testicular hemodynamics in various animal species (Fig. 1 and Table 1).

**Table 1:** Summarizing the factors that could affect testicular blood flow in farm and companion animals

Factor	Findings	Species	References
Thermal factor	• Significant increases in TBF were found in response to increases in ambient temperature	Angus bulls	Barros Adwell et al. 2018
	• Direct applying heat to the testis induced increases in TBF up to 26%	Rams	Mieusset et al. 1992
	• Warming the testes from 34 °C to 40 °C led to notable increases in the TBF	Bulls	Rizzoto et al. 2020
	• Decreases in TBF were reported during the summer season (29.43 ± 1.45 °C) compared with those found in the winter season (m 10.38 ± 1.02 °C)	Shiba bucks	Samir et al. 2018
	• No changes in the TBF were reported when the average temperature of the scrotal surface increased (> 3 °C)	Dogs	Henning et al. 2014
	• Artificial cooling of testes induced marked reductions in scrotal blood flow without any effect on TBF	Dogs	Glode et al. 1984
	• Experimental warming of the testes (to 40 °C) resulted in an extreme increase in TBF	Bulls	Rizzoto et al. 2020
	• Exposing the testes to the scrotal insulation for up to 120 h resulted in alterations in testicular temperature, the velocity of the TBF, and seminal quality	Bulls	Barca Junior et al. 2020
Seasonal factor	• Increases in TBF have been reported in spring months as compared to winter months and these alterations in TBF were coincident with sperm production (Boyd et al. 2006)	Stallions	Boyd et al. 2006; Pozor 2007
	• The highest TBF (evidenced by the lowest RI values of the supratesticular artery) was found during the breeding season (from September to March), reduced significantly from April to June. Concomitantly, testosterone, estradiol, and semen parameters were positively affected during the breeding season	Rams	Hedia et al. 2019
	• The season had a nonsignificant effect on the testicular hemodynamics	Rams	Noordhuizen-Stassen et al. 1985
	• A typical seasonal change in testicular volumes (with a maximum volume in September), testosterone levels (peak levels in December), and TBF (highest perfusion in September; the lowest RI values) all over the year	Sarda bucks	Strina et al. 2016
	• Seasonal alterations in TBF because the values of Doppler indices (RI and PI) of the testicular artery showed the greatest increases in the summer (June to August), whereas the lowest values for these indices were reported in winter (December to February). Similarly, the PI and RI values in autumn (September to November) were less than those recorded in spring (March to May),	Shiba bucks (nonseasonal animal)	Samir et al. 2018
	• Characterization of the normal blood flow of the testicular arteries over a long duration showed no statistically significant differences	Dogs	Carrillo et al. 2012
Species	• The waveforms of blood flow (either at the level of the STA or MTA) of canine, caprine, ovine, and cattle testes appear to have monophasic and non-resistive characteristics. On the opposite, TBF in stallions is often characterized by resistive-biphasic waveforms at the extent of the spermatic cord and non-resistive, monophasic frequencies from the MTA	Dogs, bucks, rams, bulls, stallions	Günzel-Apel et al. 2001; Gumbsch et al. 2002; Samir et al. 2015; Camela et al. 2019; Fadl et al. 2022; Claus et al. 2019; Rodrigues et al. 2020; Pozor and McDonnell 2004
	• It was revealed the appearance of a biphasic high-resistance blood flow within the proximal segment of the STA in dogs, similar to that observed in the STA of stallions	Dogs	Carrillo et al. 2012
Breeds	• The TBF parameters were evaluated for 334 bulls of five different breeds (Nellore, Aberdeen Angus, Hereford, Bradford, and Brangus) and showed differences among the evaluated breeds in the MV and RI of the bulls’ STA. Brangus bulls showed significantly higher values of TAMAX (16.3 ± 1.0 cm/s) as compared with the Nelore bulls (8.8 ± 0.4 cm/s). Higher RI values were found in Hereford (0.44 ± 0.01) compared with Brangus (0.36 ± 0.02) animals, whereas the PI values did not differ among breeds. In addition, there was no significant change in the PI or RI values of Nelore bulls compared with bulls of other B. taurus breeds (Angus, Bradford, Brangus, and Hereford)	Bulls	Junior et al. (2018)
	• Significantly greater values of PI and RI of the testicular arteries in bulls of the Nelore breed were found as compared with Caracu bulls	Bulls	Rodrigues et al. 2020
	• Differences in the Doppler velocimetry parameters of TBF have been found between dogs of different breeds and sizes (small dogs such as French Bulldog and large dogs such as Labrador, Rottweiler, and German Shepherd). Higher velocities (especially the EDV) of blood flow were recorded in the spermatic cord in large dogs, but the Doppler velocity values were higher at the MTA in small breed dogs	Dogs	Souza et al. 2014
Puberty or Age effect	• PSV and EDV values of the blood flow within the testicular artery were significantly lower in prepubertal dogs as compared with postpubertal dogs	Dogs	de Souza et al. 2015a
	• Age had no significant effect on TBF in stallions, however, decreased values of EDV and increased values for RI were reported in old stallions than in the middle-aged stallions in the STA region	Stallions	Pozor and McDonnell 2004
	• Decreased RI values were found at sexual maturity in rams	Rams	El-Sherbiny et al. 2022e
	• There were no significant variations in TBF Doppler indices between young and adult bulls or between pre-and postpubertal mature rams	Bulls and rams	Gloria et al. 2018; Elbaz et al. 2019
	• It was reported lower values of RI and PI of the examined STA in the mature (0.32 ± 0.04 and 0.36 ± 0.03, respectively; 2.5 years) and aged rams (0.51 ± 0.03 and 0.77 ± 0.06, respectively; 7.5 years) compared to the young ones (0.62 ± 0.03 and 0.98 ± 0.07, respectively; age 1 year)	Rams	Hedia and El-Shalofy (2022)
Bodyweight of the animal	• Greater TBF (lower Doppler indices values) was recorded in heavy horses as compared with miniature horses	Stallions	Ortega-Ferrusola et al. 2014
Sexual activity	• Increased sexual activity has a positive effect on TBF	Stallions	Ortega-Ferrusola et al. 2014
Testicular laterality	• Most studies on bucks, rams, and stallions have revealed a lack of significant differences between the right and left testes in terms of pulsed-wave Doppler indices	Bucks, rams, and stallions	Samir et al. 2015, 2018; Mandour et al. 2020; Pozor and McDonnell 2004; Camela et al. 2017; Elbaz et al. 2019
	• In Awassi rams, RI and PI were a bit increased (P ˂ 0.05) in the right testicular artery as compared with the left one	Rams	Hedia et al. 2020b
	• In dogs of various breeds and weights, the left testis is substantially larger than the right one, whereas the Doppler velocity parameters and indices did not differ between the sides	Dogs	Souza et al. 2014; de Souza et al. 2015a
Diseases and reproductive disorders	• Various diseases such as testicular torsion and varicocele in stallions and testicular tumors in dogs may induce alterations in testicular hemodynamics	Stallions and dogs	Pozor and McDonnell 2004; Pozor 2007; Bigliardi et al. 2019
The section of the artery evaluated	• The PSV, EDV, and RI values of the STA were significantly higher than those in the MTA and were higher in the MTA as compared with those in the intratesticular branches	Bulls	Gloria et al. 2018
	• Doppler indices (RI, PI) were significantly higher in the STA compared with those in the MTA	Bucks and donkey	Samir et al. 2020a, b; Gacem et al. 2020
	• Significant differences were found in Doppler parameters of TBF (PSV, EDV, RI, and PI values) measured in five regions along the testicular artery (STA [proximal, medial, and distal regions], MTA, and intratesticular branches). The RI, PI, and PSV values decreased gradually along the course of the testicular artery. The highest values were noted in the cranial segment of the STA, which decreased gradually in the other sections, whereas the lowest values were measured within the intratesticular arteries	Dogs	Carrillo et al. 2012; de Souza et al. 2015a, b; Trautwein et al. 2019
	• Morphological and hemodynamic changes were reported in different regions of the STA. Higher PSV, RI, and PI were found in the proximal region of the STA, followed by middle and distal ones. Also, significant progressive increases in the TABF and TABFR were found along the testicular cord until entering the testis	Assaf rams	Hassan et al. 2022
Psychological status of the patient	• Some animals such as dogs may experience tremors and fear, which can interfere with the Doppler examination	Dogs	Trautwein et al. 2019
Diurnal effect	• Diurnal rhythm induces significant changes in TBF over the 24 h a day	Bucks	Samir et al. 2022

Abbreviations: *TBF* Testicular blood flow; *PSV* Peak systolic velocity; *EDV* End diastolic velocity; *TAMAX* Time average maximum velocity; *RI* Resistive index; *PI* Pulsatility index; *TABF* Total arterial blood flow; *TABFR* Total arterial blood flow rate

### Environment factors

#### Thermoregulation of the testis

In most mammals, suspending the testes inside the scrotum and outside the body is to adjust the intratesticular temperature to be slightly lower than the core body temperature. Also, there is a complicated thermoregulatory system in the testis involving vascular and non-vascular components. The vascular components are the pampiniform plexus which performs countercurrent heat exchange for the warm blood entering the testis and cool blood draining from the testis (Setchell 2006; Hansen 2009), and peripheral vasodilation. Before entry to the testis, the testicular artery is more tortuous to maintain a testicular temperature of approximately 4–6 °C below body temperature, given the mechanism of countercurrent heat exchange (Waites 1970). The great extension of the testicular artery in contact with the pampiniform venous plexus favors heat exchange from arterial blood to venous blood and aids in proper thermoregulation of the testis (Brito et al. 2004). In addition, the testicular artery forms an extensive network of superficial vessels, which contribute to the mechanism of temperature regulation and dissipation of heat (Morrell 2020).

The non-vascular components of thermoregulation include physiological responses such as sweating, changes in the location of the testis relative to the abdomen, and other behavioral approaches such as seeking shade (Rizzoto and Kastelic 2020). The degree of testicular cooling is further controlled by two muscles: the tunica dartos, just beneath the skin of the scrotum, which regulates the scrotal surface area, and the cremaster muscle that controls the position of the scrotum relative to the body (Hansen 2009). Contraction of the cremaster muscle brings the testes closer to the abdomen in cold conditions while its relaxation of these permits the testes to hang away from the body in warm conditions (Morrell 2020). Furthermore, the skin of the scrotum is thin, devoid of subcutaneous fat, and has more dense sweat glands than the skin on other parts of the body contributing to heat transfer by allowing heat dissipation through sweating (Blazquez et al. 1988). The testes can thermoregulate against exposure to cold temperatures by contracting and pulling up against the body. The scrotum limits the dissipation of heat by increasing the skin rugosity to decrease the surface area exposure (Mariotti et al. 2011).

#### Impact of increased temperature on testicular function

The testicular temperature should be maintained at around 32 °C for normal spermatogenesis. Increased environment temperatures (with or without high humidity) could interfere with the thermoregulatory mechanism of the testes, disrupt the evaporative heat loss from the scrotal surface, and result in increases in the intratesticular temperature (Morrell 2020). Testes functions operate in a microenvironment close to hypoxia (Bergh et al. 2001; Barros Adwell et al. 2018). An increment in testicular heat causes increased testicular metabolism and oxygen requirements, resulting in hypoxia and the formation of reactive oxygen species, which have a significant impact on sperm production (Setchell 1978, 1998; Kastelic et al. 2017). Decreased sperm quality is primarily attributed to the effect of hypoxia and not directly to hyperthermia (Paul et al. 2009; Hamilton et al. 2016).

Exposing the testes to thermal stress via an insufficient thermoregulatory system induces a negative impact on spermatogenesis (due to its deleterious effects on all major cells within the testis), resulting in lower sperm quality and quantity characteristics (Setchell 1998; Paul et al. 2008; Hansen 2009; Hedia et al. 2020a; Shahat et al. 2020); this has more drastic consequences for human and animal reproduction and population. In deed, influences of increased testicular temperature depend on the extent and duration of testicular heating. Mild increases in testicular temperature may cause only a temporary reduction in sperm quality, and prolonged and/or substantial heating are likely to cause infertility. Furthermore, a severe thermal insult may cause permanent cessation of spermatogenesis (Rizzoto and Kastelic 2020).

The inability of the animal to adapt (through different physiological and behavioral mechanisms and trials) to the increased environment temperature results in heat stress. When assessing the impact of heat stress, the temperature humidity index (THI) should be addressed, together with the effect of season, even in temperate climates (Llamas-Luceño et al. 2020). The effects of THI on the semen characteristics of bulls were found to be breed-dependent (Gloria et al. 2021). For instance, significant reductions in the semen volume, sperm concentration, total sperm in the ejaculate, total sperm motility, sperm membrane integrity, and sperm normal morphology by an increasing THI were found in the Belgian Blue bulls, but not in Brown Swiss bulls (Gloria et al. 2021). These variations could be attributable to differences in anatomy, endocrinology, or resilience of this breed after a period of high-temperature exposure. Therefore, special husbandry strategies including ventilation, shading, antioxidants supplementation, or the timing of semen collection (Morrell 2020; Shahat et al. 2020) should be considered to alleviate the negative impact of heat stress, especially in some breeds of beef bulls such as Belgian Blue bulls. Also, breeds should be selected according to climatic conditions and the rearing purpose of livestock (Morrell 2020).

Regardless of the etiology, increased scrotal/testicular temperature could not initially deteriorate sperm morphology (for a period known as an epididymal transit time), while its negative impacts are noticed later (Kastelic and Rizzoto 2021). However, high percentages of epididymal sperm abnormalities were found when collected soon after scrotal thermal exposure (Kastelic et al. 1996). Another study (Vogler et al. 1991) found alterations in the epididymal sperm quality only following cryopreservation. Older bulls showed high sensitivity to THI at spermatogenesis compared with semen collection, exhibiting more than three times higher negative effects on cryopreserved sperm quality (Llamas-Luceño et al. 2020). In general, sperm parameters usually return to pre-treatment standards within approximately six to eight weeks after the thermal stress exposure (Llamas-Luceño et al. 2020; Kastelic and Rizzoto 2021). However, a prolonged and/or severe increase in testicular temperature will increase the interval for the return of sperm quality (Kastelic and Rizzoto 2021). Therefore, using colour Doppler ultrasonography to measure testicular hemodynamics is critical for estimating the impact of high ambient temperature on testicular functions.

#### Thermal factors or increasing environment temperature on TBF

Regardless of the method of assessment, this chapter is illustrating the effect of the thermal factor or increasing environment temperature on TBF in various animal species. Numerous studies on domestic animals (bulls, rams, bucks, and dogs) have evaluated the changes in TBF in response to changes in testicular or ambient temperature. In Angus bulls, significant increases in TBF were found in response to increases in ambient temperature (from 5 °C to 35 °C, increases were observed in blood flow [2.45 versus 4.23 mL/min/100 g testis] as well as increases in testicular temperature [31.8 °C versus 34.9 °C]; Barros Adwell et al. 2018). By directly applying heat to the testis in rams, the testicular temperature increased to 36 °C and blood flow (outside the scrotum) increased by up to 26% (Mieusset et al. 1992). In addition, in anesthetized bulls (Rizzoto et al. 2020), warming the testes from 34 °C to 40 °C led to notable increases in the TBF (13.2 ± 2.7 versus 17.7 ± 3.2 mL/ min/100 g of testis), oxygen extraction (31.2% ± 5.0% versus 47.3% ± 3.1%), and oxygen consumption (0.35 ± 0.04 versus 0.64 ± 0.06 mL/ min/100 g of testis).

The response of TBF to thermal factors also depends on the extent of the increases in testicular temperature relative to the body temperature. For example, in bucks, changes in the TBF between seasons were coincidental with the variations in ambient temperature (Samir et al. 2018). Decreases in TBF were reported during the summer season (mean maximum temperature 29.43 ± 1.45 °C) compared with those found in the winter season (mean maximum temperature 10.38 ± 1.02 °C) (Samir et al. 2018). These results disagreed with the findings of previous studies. The reason for this discrepancy might be whether the testicular temperature surpassed the body temperature or not. As previously proposed (Setchell 1978), blood flow in sheep testis is increased when the testicular temperature is higher than the body temperature, but there is no increase in TBF when the temperature increases up to the body temperature. Therefore, the seasonal coincidental variations in TBF found in bucks might be a component of the offsetting mechanism of goat testes for the control of testicular blood flow as a way to overcome the variations in ambient temperature to maintain normal testicular function (Samir et al. 2018).

The effect of thermal factors on testicular hemodynamics may depend on the species. One study in dogs found no changes in the TBF when the average temperature of the scrotal surface increased (> 3 °C) (Henning et al. 2014). Also, after the artificial cooling of dog testicles, marked reductions in scrotal blood flow was observed without any effect on the blood flow to the testis (Glode et al. 1984). The effect of a thermal factor on the testis might not be limited to the TBF. For example, Waites et al. (1973) reported that when the scrotum of mice was immersed in water at a temperature ranging from 28 °C to 45 °C for 20 min, increases were observed in the blood flow within the scrotum and in the brains of anesthetized mice. These findings demonstrate the species' effect on TBF in response to increased testicular temperature.

Nevertheless, breed type might also influence TBF in animals in response to thermal factors. For example, increased scrotal temperature did not induce changes in TBF in rams selected for skin wrinkling, but it did increases the TBF in rams that were not selected for skin wrinkling (Fowler and Setchell 1971). Furthermore, exposing bulls to high ambient temperatures resulted in a substantial effect on spermatogenesis in *Bos taurus* bulls, whereas *Bos indicus bulls* were less severely affected (Johnston et al. 1963; Skinner and Louw 1966).

It is worth mentioning that the differences between the continental or British breed (*B. taurus*) bulls and the Indian or Zebu breed (*B. indicus*) bulls for either susceptibility or adaptability to the deleterious effects of increased testicular temperature (due to warm environments) on sperm quality may be attributable to various morphological and anatomical differences in the testicular artery (Nichi et al. 2006). The wall of the testicular artery of *B. indicus* bulls is thinner, and there is a shorter distance between the arterial and venous blood in the testicular vascular cone (Brito et al. 2004). Furthermore, bulls in this breed have more functional sweat glands with a larger perimeter (Carvalho et al. 1995) in the scrotal skin, a larger pendulous scrotum (Brito et al. 2004; Siqueira et al. 2012), and a higher length of the testicular artery compared with other breeds (Brito et al. 2003, 2004). These morphological changes may contribute, in part, to the greater resistance of *B. indicus* bulls to high environmental temperatures by conferring better TBF and facilitating heat exchange between the testicular artery and veins (Kastelic et al. 1997).

Recently, experimental warming of the testes (to 40 °C) in both *B. taurus* and *B. indicus* bulls resulted in an extreme increase in TBF to provide enough oxygen in a way to meet the increased metabolic demands of the testes and avoid hypoxia (Rizzoto et al. 2020). Exposing the testes to the scrotal insulation for up to 120 h resulted in alterations in testicular temperature, the velocity of the TBF, and seminal quality in bulls (Barca Junior et al. 2020). Therefore, in-depth analyses should also be directed to the nature of the thermal stress to which animals are exposed and consideration given as to whether the thermal factor is restricted to the testes (i.e., local effect by warming the testis or insulation) or is a general effect resulting from exposing the animal to the high environment or experimental temperature (El-Sherbiny et al. 2022c).

#### Effect of season and climatic changes on TBF

Several studies highlighted the impact of climatic changes on semen quality and the fertilizing capacity of males in various domestic animals (Pérez and Mateos 1996; Boyd et al. 2006; Samir et al. 2018; Hedia et al. 2019) with contradictory findings, perhaps because the effect of the season can vary greatly among climatic zones (Llamas-Luceño et al. 2020) and geographic location (Pérez and Mateos 1996). On the opposite, little attention has been paid to the effect of seasonality on TBF. In stallions, increases in TBF have been reported in spring months as compared to winter months (Boyd et al. 2006; Pozor 2007). Increased TBF during the breeding seasons of stallions was coincident with increases in sperm production (Boyd et al. 2006).

In fat-tailed rams (Hedia et al. 2019), RI values of the supratesticular artery were lowest during the breeding season (from September to March) and increased significantly in April (up to 50%) reaching the highest value during June. Concomitantly, testosterone, estradiol, and semen parameters were positively affected during the breeding season. In addition, high negative correlations were found between values of RI and PI and sperm concentration and progressive motility (Hedia et al. 2019). Recently, RI values of TBF showed high negative correlations with the curvilinear velocity and linearity (both slow and rapid defaults) of spermatozoa as assessed by computer-assisted sperm analysis (CASA) during the breeding season of Polish Heath rams (Kozłowska et al. 2022). Also, the advanced features of higher sperm abnormalities (Grade 4 of the motile sperm organelle morphology examination; MSOME as detected by CASA) were positively correlated with RI and PI values before the breeding season (0.61, 0.52, respectively), and after the breeding season (0.60, 0.46, respectively). Concurrently, high-quality semen was derived during the breeding season, whereas high sperm abnormalities (related to sperm cells' DNA vacuolization and fragmentation) were noticeable before the breeding season. However, an old study on rams showed a nonsignificant effect of season on testicular hemodynamics (Noordhuizen-Stassen et al. 1985).

In a 12-month study in Sarda bucks (breed with seasonal variations of gonad activity under natural daylight conditions), Strina et al. (2016) showed a typical seasonal change in testicular volumes and testosterone levels with a maximum testicular volume in September and peak testosterone levels in December. Concomitantly, TBF follows the seasonal changes in the testicular parenchyma depending on the functional activity of the testis, and the lowest RI values were observed in September. Likewise, in nonseasonal breed bucks such as Shiba bucks, distinct seasonal fluctuations in TBF were noted, especially for Doppler indices (Samir et al. 2018). The greatest increases in the values of the RI and PI of the testicular artery were found in the summer (June to August). Similarly, the PI and RI values in autumn (September to November) were less than those recorded in spring (March to May), whereas the lowest values for these indices were reported in winter (December to February) (Samir et al. 2018). However, in dogs, characterization of the normal blood flow of the testicular arteries (PSV, EDV, RI, and PI) over a long duration showed no statistically significant differences (Carrillo et al. 2012). TBF variations between seasons are most often attributed to differences in the testicular endocrine and spermatogenic functions (Joffre and Joffre 1973) and may be the cause of seasonal changes in testicular function (Boyd et al. 2006; Pozor 2007). Therefore, some treatments such as pentoxifylline treatment in stallions (Pozor et al. 2011), FSH administrations in bucks (Samir et al. 2023), and curcumin supplementations in rams (El-Sherbiny et al. 2022d), should be addressed because they modulate TBF and improve the fertilizing capacity of males during the non-breeding seasons.

Seasonal breeders such as cats (Alexandre-Pires et al. 2012) deer (Wagener et al. 2010; de Souza Cunha et al. 2019), and hamsters (Mayerhofer et al. 1989) showed variations in testicular mass and TBF throughout the year. Also, they exhibited seasonal activity of angiogenesis and variations in the histomorphology and the cellular composition of the testis (Pyter et al. 2005). Angiogenesis defines as the development of new blood vessels from differentiated endothelial cells (Alessi et al. 2004). Angiogenesis may be found inside the testicular tissues during the process of spermatogenesis and spermiogenesis (Lecouter and Ferrara 2002). Changes in the angiogenesis process play a crucial role in the vascular growth and regression of the testes during the breeding and nonbreeding seasons. Testicular angiogenesis is known to increase during testicular recrudescence in seasonal breeders such as the hamster (Mayerhofer et al. 1989) or to decrease in response to feeding restrictions in rabbits (Carvalho et al. 2009). In the hamster, there are seasonal variations in the permeability of the testicular microvasculature concurrent with the gonadal activity (testis mass and sperm production) (Mayerhofer et al. 1989). In male cats, testicular vascularisation appears to be predominantly increased in three photoperiod windows of time (November/December, March/April, and June/July) (Alexandre-Pires et al. 2012). These seasonal alterations might be attributable to the circadian oscillators to the timing of light exposure and could influence their reproductive performance (Alexandre-Pires et al. 2012).

Sexually mature Roe deer bucks (*Capreolus capreolus*) showed a complete arrest in spermatogenesis in the winter months, being started later in the spring and reaching the highest peak during the breeding season (May to September) (Blottner et al. 2006; Wagener et al. 2010). In addition, it showed prominent reductions in testicular size and decreased function in the post-rutting period (Elmi et al. 2020). Such patterns in the seasonal testicular cycle could be valuable and give potentially information that could be important to other species and further studies may be required.

In seasonal breeders such as Roe deer, there is a seasonal expression pattern of vascular endothelial growth factor (VEGF), being the highest level at the peak of spermatogenesis during the pre-rutting season and reaching the lowest level at the end of the rutting season **(**Wagener et al. 2010). The vascular endothelial growth factor is an important proangiogenic factor responsible for vascular dilatation and increases vascular permeability (Dvorak et al. 1999; Conway et al. 2001). VEGF and its receptors are expressed in all testicular cells, both germline and interstitial components (Liu and Yang 2004), and play a pivotal role in male germ cell differentiation, proliferation, and migration (Sargent et al. 2016). It has a great role in regulating germ cell survival in the bovine testis during the spermatogenesis cycle (Caires et al. 2009). These findings may suggest the potential roles of VEGF in the regulation of spermatogenesis but may not be participated predominantly in the microvasculature of the testis. Other growth factors are also incorporated into the seasonal variability of testicular angiogenesis such as transforming growth factors, fibroblast-like growth factors, and insulin-like growth factors (Wagener et al. 2003, 2005).

In addition to its potential roles in the regulation of germ cell differentiation and migration, and blood vessel development, VEGF plays an important role in the integrity of the blood-testis barrier (BTB) (Reddy et al. 2012). The BTB is one of the tightest blood-tissue barriers in the mammalian body. It divides the seminiferous epithelium into basal and adluminal compartments. Thus, the BTB regulates the entry of nutritional and vital substances (e.g. sugars, amino acids, hormones, electrolytes) and harmful toxicants (e.g., environmental toxicants, drugs, chemicals) into the apical compartment of the seminiferous tubules (Cheng and Mruk 2012). This role creates a special microenvironment for the seminiferous tubule (being not penetrated by blood vessels, lymph vessels, or nerves) for normal postmeiotic germ cell development (i.e., spermiogenesis and spermiation) occurrence. Therefore, the BTB is considered an immunological barrier that is necessary for the differentiation of spermatogonia during spermatogenesis.

Because TBF has an important role in the regulation of testicular temperature, exposing the testis to thermal factors could deteriorate the cellular components of the testis including Sertoli cells which may affect the permeability of the BTB and impair its pivotal roles in the spermatogenesis process. Exposing the testis to hypoxia significantly suppressed the proliferation of Sertoli cells, induced cellular apoptosis, and could damage the integrity of BTB and spermatogenesis of the testis (Hao et al. 2013). In total, the deterioration of BTB could generate anti-sperm antibodies which bind to sperm parts and reduce the fertilizing capacity of sperm. Other causes such as testicular trauma, Cadmium toxins, and chemotherapeutic drugs using nanotechnology for cancer therapies could negatively affect the integrity of the BTB and impair spermatogenesis (Cheng and Mruk 2012).

### Genetic factors

#### Species effect

There are species differences in the characterization of TBF in farm and companion animals (Supplement Tables 1 & 2). Previous literature showed that waveforms of the blood flow within the testicular artery (either at the level of STA or at the MTA) of humans (Middleton et al. 1989), canines (Günzel-Apel et al. 2001; Gumbsch et al. 2002), feline (de Brito et al. 2015), caprine (Samir et al. 2015), ovine (Camela et al. 2019; Fadl et al. 2022), and cattle (Claus et al. 2019; Rodrigues et al. 2020) testes appear to have a monophasic, nonresistive character. Blood flow to the testes in stallions, on the other hand, is often characterized by resistive-biphasic waveforms at the extent of the spermatic cord (Bollwein et al. 2008) and nonresistive, monophasic frequencies from the MTA (Pozor and McDonnell 2004). The horizontal pattern of the longitudinal direction of the testes, its proximity to the body wall, and the proportionally short spermatic cord with the convoluted artery account for the resistive appearance of waveforms at the twisted part of the testicular artery in stallions (Pozor and McDonnell 2004). Interestingly, one study in dogs (Carrillo et al. 2012) revealed the appearance of a biphasic high-resistance blood flow within the proximal segment of the STA, similar to that observed in the STA of stallions (Pozor and McDonnell 2004).

#### Effect of breeds

Within each species, the effect of breed on TBF has received little attention. Little studies have been performed on bulls and dogs to illustrate the effect of breeds on testicular hemodynamics. Junior et al. (2018) evaluated the TBF parameters (MV, RI, and PI) of 334 bulls of five different breeds (Nellore, Aberdeen Angus, Hereford, Braford, and Brangus) and showed differences among the evaluated breeds in the MV and RI of the bulls’ STA. Brangus bulls showed significantly higher values of TAMAX (16.3 ± 1.0 cm/s) as compared with the *Nelore* bulls (8.8 ± 0.4 cm/s). The researchers also observed higher RI values in Hereford (0.44 ± 0.01) compared with Brangus (0.36 ± 0.02) animals, whereas the PI values did not differ among breeds. In addition, there was no significant change in the PI or RI values of *Nelore* bulls compared with bulls of other *B. taurus* breeds (Angus, Bradford, Brangus, and Hereford) (Junior et al. 2018). A recent study showed significantly greater values of PI and RI of the testicular arteries in bulls of the *Nelore* breed as compared with *Caracu* bulls (Rodrigues et al. 2020). These results could be attributed to variations in the shape and size of the testis between the breeds, which could result in variations in testicular hemodynamics. The negative correlations between PI and RI values and testicular length and scrotal circumference observed in jackasses (Gacem et al. 2020) and bulls (Rodrigues et al. 2020) could support this hypothesis.

Differences in the Doppler velocimetry parameters of TBF have been found between dogs of different breeds and sizes (small dogs such as French Bulldogs and large dogs such as Labrador, Rottweiler, and German Shepherd) (Souza et al. 2014); namely, higher velocities (especially the EDV) of blood flow were recorded in the spermatic cord in large dogs, but the Doppler velocity values were higher at the MTA in small breed dogs. Doppler examination of the intratesticular blood vessels showed nonsignificant differences in the velocity parameters among the studied breeds.

### Physiological and other factors

#### Effect of puberty or age on TBF

There is a lack of studies scrutinizing the effect of age on TBF in domestic animals. Most studies revealed increases in TBF with age. PSV and EDV values of the blood flow within the testicular artery were significantly lower in prepubertal dogs as compared with postpubertal dogs **(**de Souza et al. 2015a). Age had no significant effect on TBF in stallions, however, decreased values of EDV and increased values for RI were reported in old stallions than in the middle-aged stallions in the STA region (Pozor and McDonnell 2004). Also, several reports on humans (Middleton et al. 1989; Oyen 2002) reported lower RI values in postpubertal ages (0.6) than in those of prepubertal ages (0.87). Decreased RI values at sexual maturity in rams (El-Sherbiny et al. 2022e) are associated with vasodilation and increased blood flow to the testis. In the latter, increased TBF with age may be attributed to the physiologic hypovascularization of the testes (Saunders et al. 1998; Dudea et al. 2010). In contrast, there were no major variations in the values of TBF Doppler indices between young and adult bulls (Gloria et al. 2018) or between pre-and postpubertal mature rams (Elbaz et al. 2019).

Recently, Hedia and El-Shalofy (2022) reported lower values of RI and PI of the examined STA in the mature (0.32 ± 0.04 and 0.36 ± 0.03, respectively; 2.5 years) and aged rams (0.51 ± 0.03 and 0.77 ± 0.06, respectively; 7.5 years) compared to the young ones (0.62 ± 0.03 and 0.98 ± 0.07, respectively; age 1 year).

#### Effect of testicular size, and the bodyweight on TBF

Only a few studies have evaluated the effect of the testicular volume and body weight of animals on TBF. It was hypothesized that having a greater testes volume is linked to having a lower RI value and in turn higher TBF (Paltiel et al. 1994). In addition, greater blood perfusion (lower Doppler indices values) was recorded in heavy horses as compared with miniature horses (Ortega-Ferrusola et al. 2014). Therefore, it is of interest for the technician to consider various parameters that could affect testicular hemodynamics and to identify the number of measurements needed to attain better measurement accuracy (Pozor and McDonnell 2004).

#### Effect of sexual activity on TBF

To obtain a proper assessment of TBF in domestic animals, the sexual activity of the male is a very important factor that should be addressed. In stallions, the researchers reported the positive effect of increased sexual activity on the improvement of TBF (higher velocities and lower RI of the testicular artery) (Ortega-Ferrusola et al. 2014). Increased blood perfusion to the testis after sexual activity or ejaculation may be due to the increase in the demand for more sperm production by the testicular tissues, the stallions' constant exposure to mares, as well as changes in the social climate (Ortega-Ferrusola et al. 2014). Ortega-Ferrusola et al. (2014) evaluated the effects of 12 months of frequent semen collection on TBF. However, this study did not have a control group, which makes this observation questionable. For that reason, researchers have recommended a sexual rest (no ejaculation) before the examination of blood flow to the prostate and the testis with power or pulsed Doppler ultrasonography, because Doppler parameters could be influenced by ejaculation (Alonge et al. 2018a, b). Ejaculation is an important factor that could affect testicular hemodynamics because it could induce increases in body temperature and peripheral vasoconstriction. However, previous literature in rams (Gouletsou et al. 2003; Ahmadi et al. 2012) found no significant effect of ejaculation on testicular echotexture, and further investigation on its effect on testicular hemodynamics is needed.

#### Effect of testicular laterality

Most studies on bucks (Samir et al. 2015, 2018, 2020a, b; Mandour et al. 2020) and stallions (Pozor and McDonnell 2004) have revealed lack of significant differences between the right and left testes in terms of pulsed-wave Doppler indices. Similar results were obtained in Dorper rams (Camela et al. 2017) and Barki Egyptian rams (Elbaz et al. 2019). In Awassi rams, however, RI, and PI, were a bit increased (P ˂ 0.05) in the right testicular artery as compared with the left one (Hedia et al. 2020b). These differences might be related to the asymmetry in the volume or the orientation between the right and left testes. The evaluation of testicular size in dogs of various breeds and weights revealed that the left testis is substantially larger than the right (Souza et al. 2014), whereas the Doppler velocity parameters and indices did not differ between the sides (Souza et al. 2014; de Souza et al. 2015a).

#### Diseases and reproductive disorders

Various diseases, especially fever, may induce alterations in testicular hemodynamics owing to increased body temperature. Decreases in testicular microcirculation, testicular damage, and increased ROS activity were reported in systemic arterial hypertensive rats (Colli et al. 2019). Color Doppler ultrasonography has been used to diagnose different testicular affections in animals species, such as testicular torsion and varicocele in stallions (Pozor and McDonnell 2004; Pozor 2007) and testicular tumors in dogs (Bigliardi et al. 2019). Dogs and stallions are commonly susceptible to testicular torsion due to the anatomical characters of the spermatic cord and the testicular orientation (Edwards 2008; Samir et al. 2021; Raisi and Davoodi 2022). Dilation of the spermatic cord vessels, alterations in TBF and echogenicity of testicular parenchyma, and colic symptoms are observed in cases of testicular torsions based on the severity of the torsion (Samper et al. 2007; Raisi and Davoodi 2022). Other than this, color Doppler imaging has been used over the past 20 years in numerous studies to characterize TBF and to assess its relationship to the testicular function and semen quality in various animal species such as bulls (Gloria et al. 2018; Junior et al. 2018), stallions (Pozor and McDonnell 2004; Pozor 2007; Pozor et al. 2014; Ortega-Ferrusola et al. 2014; Ortiz-Rodriguez et al. 2017), rams (Batissaco et al. 2013; Camela et al. 2017, 2019; Ntemka et al. 2018; Hedia et al. 2019, 2020a), bucks (Samir et al. 2015, 2018, 2020a, b; Strina et al. 2016; Mandour et al. 2020), and dogs (Gumbsch et al. 2002; Zelli et al. 2013; de Souza et al. 2014, 2015a, b; England et al. 2017; Bigliardi et al. 2019; Lemos et al. 2020).

 Indeed, evaluation of the semen quality and its fertilizing capacity are the golden standard for assessing testicular functions in farm and companion animals and very crucial to proving animal fertility. However, the evaluation of TBF sheds light on the reproductive potentials of males and is considered a good, rapid, and non-invasive tool for diagnosis and monitoring many infertility problems. Hence, Doppler ultrasonography does not represent a front-line diagnostic tool for specific reproductive disorders-rather it provides indirect clues by detecting alterations in testicular hemodynamics, with extra evidence for some conditions provided by other important methods of assessment such as semen quality (Samir et al. 2021).

### Technical factors affecting ultrasonographic Doppler estimation of TBF

#### Effect of the section of the artery evaluated

**Fig. 2:**
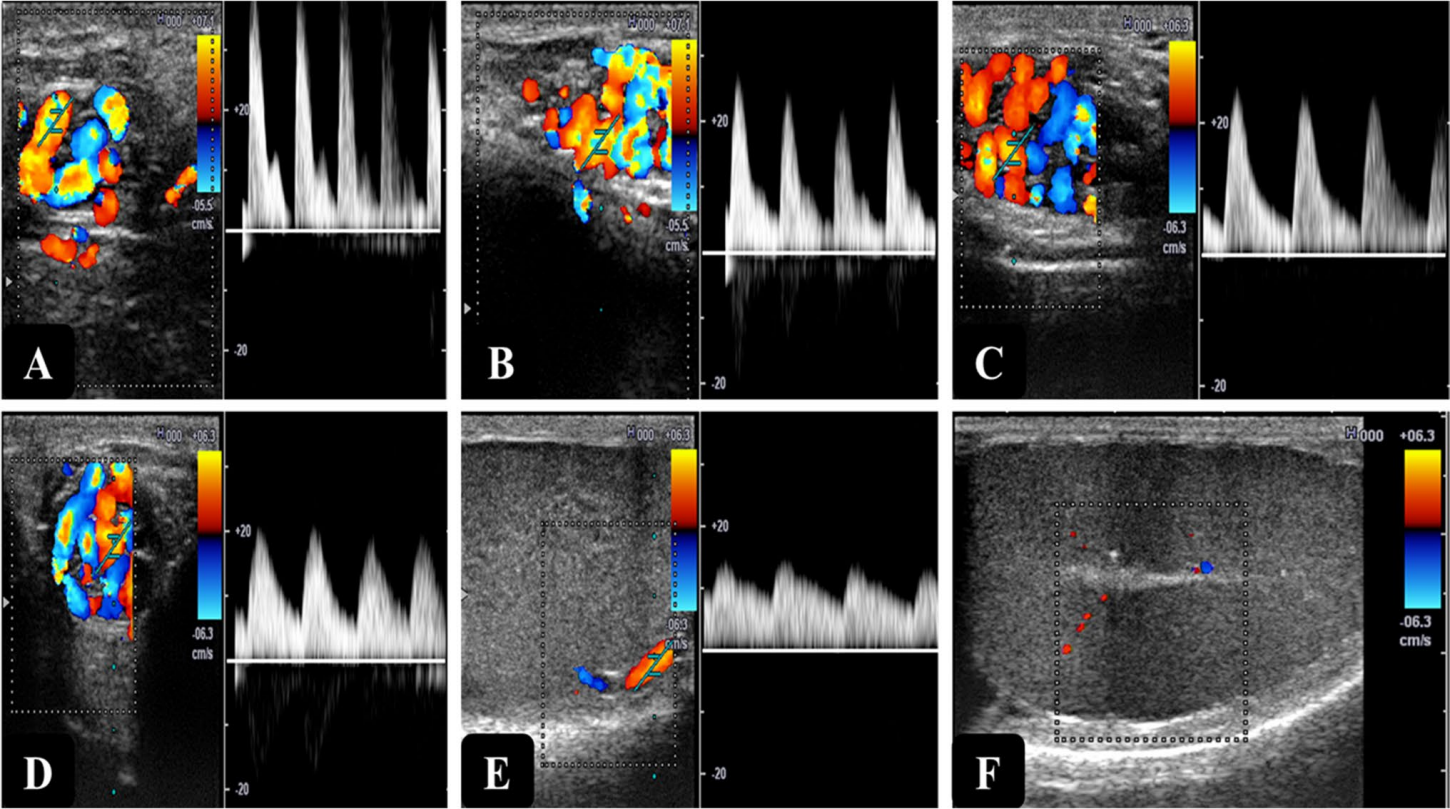
Variable characteristics features of testicular blood flow in different portions of the testicular arteries in bucks. Notice: when the examined segment is approaching the testis, the waveforms of blood flow are less resistive (**A**: the initial segment of the supratesticular artery (STA), **B**: the middle segment of the STA, **C** & **D**: lower segments of the STA, **E**: marginal testicular artery, **F**: intratesticular artery)

Because characterizations of TBF are significantly affected by the section of the vessel examined by color Doppler ultrasonography, one of the most important issues to address is the identification of the segment of the testicular artery to be examined (Fig. 2). In bulls, the PSV, EDV, and RI values of the STA were significantly higher than those in the MTA and were higher in the MTA as compared with those in the intratesticular branches (Gloria et al. 2018). Similarly, in Shiba bucks (Samir et al. 2020a, b) and donkeys (Gacem et al. 2020), Doppler indices (RI, PI) were significantly higher in the STA compared with those in the MTA. In stallions, mean RI values in the MTA were just marginally lower than in the convoluted part (Pozor and McDonnell 2004).

Even within the same individual, blood flow velocities (PSV and EDV) measured at the STA were variable because of the convoluted course of the vessel at this section (Gloria et al. 2018). Therefore, researchers have preferred using Doppler indices of TBF because they are highly repeatable within the same individual and between the examinations because they are not angle-dependent. In llamas, assessment of TBF within the STA was more useful for determining the infertile males, because PSV and EDV of the STA differed between infertile and fertile males (Kutzler et al. 2011). Doppler ultrasonography cannot assess fertility, but it assesses TBF. However, altered TBF affects testicular functions, thus the fertility status is also negatively affected. However, MTA is a preferred section for examining the TBF than those at the STA by many authors, due to the best accessibility and a straight course of this part of the vessel, thus facilitating the detection and obtaining reproducible Doppler measures of TBF as reported in dogs (Günzel-Apel et al. 2001). Intratesticular arteries could be seen as colour pixels of various sizes in any ultrasonographic examination; however, they made pulsed-wave Doppler measurement difficult (Günzel-Apel et al. 2001), and the power Doppler technique may be helpful for proper assessment. The newer ultrasound machines with a high-frequency transducer (for example 10 MHz) allow for obtaining these parameters from intratesticular arteries in bulls (Gloria et al. 2018), dogs (Gloria et al. 2020), and rams (Hedia and El-Belely 2021).

In addition, the characterization of the normal testicular hemodynamics showed changes in the course of the testicular artery. For example, in Beagle dogs, a flow pattern of a highly resistive vessel was observed at the proximal portion of the STA, whereas in the looping portion of the STA, MTA, and intratesticular vessels, the flow experienced a reduced resistance pattern (Carrillo et al. 2012; Trautwein et al. 2019). In more descriptive studies in dogs (Carrillo et al. 2012; de Souza et al. 2015a, b; Trautwein et al. 2019), significant differences were recorded in Doppler parameters of blood flow (PSV, EDV, RI, and PI values) measured in five regions along the testicular artery (STA [proximal, medial, and distal regions], MTA, and intratesticular branches). The RI, PI, and PSV values decreased gradually along the course of the testicular artery. The highest values were noted in the cranial segment of the STA, which decreased gradually in the other sections, whereas the lowest values were measured within the intratesticular arteries (This information can be seen in Fig. 2).

The fact that the testicular artery arises exclusively from the aorta, a vessel with high resistance to blood flow, may explain increased velocities at the STA relative to other segments near the entry of the testis and the intratesticular branches (Trautwein et al. 2019). The prolongation and tortuosity of the artery results in a decrease in its resistivity and the thickness of the vascular endothelium is reduced as it approaches the testis. As the vessel course enters the testis, the RI and PI values decrease. These differences may be due to differences in the morphological and hemodynamic characteristics of the testicular artery during its course in the spermatic cord and the testicles. Previous studies reported decreases in the thickness of the artery wall and the vessel lumen as it enters the testis (de Souza et al. 2014; Souza et al. 2014). Similarly, a recent study in Assaf rams (Hassan et al. 2022) reported morphological and hemodynamic changes in different regions of the STA. The study showed higher PSV, RI, and PI in the proximal region of the STA, followed by middle and distal ones. Also, significant progressive increases in the TABF and TABFR were found along the testicular cord until entering the testis. Another aspect that would support this reduction in blood flow velocity as the vessel reaches the testis is the increased vascular dichotomization that occurs when the artery branches as it enters the testicular parenchyma, dissipating blood velocity and favoring gas exchange with the tissue (Trautwein et al. 2019).

The testis receives its blood supply mainly from the testicular artery which has a coiled appearance before entering the testis (at the pampiniform plexus, PFP). TBF at this portion is low (evidenced by lowered RI and PI values compared to other segments) as reported in various animals species such as jackass (Gacem et al. 2020), dogs (Trautwein et al. 2019), and rams (Hassan et al. 2022). Reducing the amount of blood that enters the testis is very crucial to reduce the testicular temperature by about 4–5 C below the body temperature (Lloyd-Jones et al. 2015). Also, it reduces the tissue oxygen pressure (hence, the testis operates its function in a microenvironment close to hypoxia that could be helpful to protect sperm DNA from the damage caused by free oxygen radicals) which facilitates a steady and consistent blood flow for proper functioning (Gacem et al. 2020). Since the blood flow velocity measures differed between the measured sections/segments along the spermatic cord in dogs, it is critical to correctly classify the measured area, as even a few cranial centimeters can have a positive impact on the measured blood flow velocity (Trautwein et al. 2019).

Collectively, to prevent any incorrect measurements or misdiagnoses, the operator must be conscious of the region being tested (Trautwein et al. 2019; Gacem et al. 2020) and obtain good references for testicular hemodynamics for better detection and monitoring of reproductive system dysfunction.

#### Effect of the psychological status of the patient on TBF

Defining the Doppler angle from the testicular arteries is difficult because of their tortuous anatomy (especially at the STA). Therefore, performing the pulsed-wave Doppler examination in a closed, calm room with temperature control is recommended in order to reduce animal motion and breathiness (Pozor 2007). Additionally, assessing the animals prior to feeding and exercising can help to prevent unnecessary visceral movement (Araujo and Ginther 2009).

Some animals (such as dogs) may experience tremors and fear, which can interfere with the examination. Doppler ultrasonography can be facilitated using blankets to leave only the scrotum on display and having the assistant caress and talk to the dogs during the assessment can provide comfort and animal welfare (Trautwein et al. 2019). Tachycardia can occur in stressed and/or anxious animals, which can affect the morphology of the Doppler waveform (Araujo and Ginther 2009; Trautwein et al. 2019). In some cases, sedation of nervous animals is important; it does not affect the blood flow of the genital organs and facilitates scanning (Araujo and Ginther 2009). However, some animals such as stallions may be able to stand quietly for enough time when simultaneously fed with a little grain or hay (Pozor 2007).

#### Doppler setting and procedures

Because of the tortuous appearance of the testicular artery, Doppler examination is considered to be time-consuming and requires the patience and technical skills of the operator as well as the cooperation of the animal. Therefore, several points listed below are practical recommendations that should be implemented before and during the spectral Doppler examination of testicular arteries in domestic animals.

##### Type of transducer (linear or convex)

A linear transducer with a broad range of frequencies (between 5–7.5 MHz) is most useful for evaluating scrotal contents, including the vasculature (such as STA) at the inguinal area between the hind limbs. Other convex transducers can be used; however, manipulation is more difficult in the tight, inguinal area (e.g., in the stallion) (Ginther and Utt 2004; Pozor and McDonnell 2004; Pozor 2007). A micro convex transducer could provide a wider variety of angles of the ultrasound beam, which helps in obtaining optimal insonation for spectral analysis. Furthermore, for the evaluation of TBF inside the MTA and arteries in the testicular parenchyma, a transducer with greater frequencies (7.5–17 MHz) may be preferable. Low blood flow could be evaluated more frequently with Power Doppler, particularly in vessels within the testicular parenchyma (Ginther and Utt 2004).

##### Adjusting the angle of the Doppler (better insonation)

The best insonation for spectral Doppler ultrasonography is when the direction of the blood flow is parallel to the ultrasound beam. However, since this is rarely possible, an "angle correction" may need to be used to obtain accurate velocimetric measures of TBF (Ginther 2007; Pozor 2007). To improve the spectral analysis of the blood flow, the operator should manipulate the position of the probe and use the angle correction mode to improve insonation. Usually, a Doppler angle of 30° to 60° is required (Pozor and McDonnell 2004; Ginther 2007; Pozor 2007) to produce an accurate spectrum of TBF. Alternatively, with newer machines, an ultrasound beam angle can be modified to become parallel to the blood flow direction. Doppler indices such as RI are not angle-dependent and can be taken without worrying about the insonation. The sample gate size (about 1–2 mm in small ruminants and dogs and 2–4 mm in large domestic animals) is centered on the portion of the artery that will be analysed and spans the entire vessel's width (Ortega-Ferrusola et al. 2014).

##### Fixed Doppler settings

For research purposes, Doppler velocimetric measurements should be obtained by the same operator and using the same settings of the ultrasonographic equipment to avoid interobserver and interdevice variations (Ginther 2007). In addition, to avoid any possible changes caused by the subject’s circadian cycle, it should be performed at the same time of day (Zaidi et al. 1995; Pozor 2007).

##### Evaluator experience

Obtaining good images of color pulsed-wave Doppler also depends on the experience and patience of the technician with a good ultrasound device. Moreover, measuring blood flow velocity requires some anatomical knowledge and vessel orientation. Evaluating the blood flow along the entire section of the testicular artery requires adequate time (Trautwein et al. 2019; Samir et al. 2021). The longest time may be required to examine the intratesticular sections with an average measurement time requirement of 15–20 min. However, it is questionable whether obtaining all Doppler parameters from each portion of the testicular artery is necessary or not to get an accurate diagnosis of TBF in various animal species. The MTA is suggested earlier by more than one author as the best segment to assess TBF correctly (Ginther and Utt 2004; Gloria et al. 2018; Samir et al. 2021). Importantly, the identification of MTA and intratesticular artery in cats are difficult, perhaps due to the small testicular volume (Brito et al. 2015, 2018).

## Conclusion and challenges

TBF plays an important role in animal reproduction, and its assessment is considered a consistent part of health management in males. Color Doppler ultrasonography is proven to be a safe and non-invasive aid for the assessment of TBF. Estimating the blood flow at the MTA section using the color-pulsed Doppler ultrasonography is the best approach, at this moment, to assess testicular hemodynamics in various farm and domestic animals under clinical circumstances. On the contrary, many factors could influence testicular hemodynamics such as environment (thermal and seasonal effects) and physiological (species, breeds, age, size, body weight, and sexual maturity) factors. Effects of breed or species on TBF could be related to the variations in the shape, size, orientation, and other anatomical characteristics of the testes, scrotum, and vasculatures. Also, differences observed in the testicular hemodynamics within the same breed or even within individuals may result from some technical aspects (segments of the evaluated vessel and technique). These factors can significantly modify the TBF and thus should be considered when establishing reference values in domestic animals for better clinical diagnosis and to ensure correct assessments. Further studies within each species that include a large number of animals should be conducted to investigate the many scientific issues concerning the validity of this technique in animal reproduction practice.

## Supplementary Information

Below is the link to the electronic supplementary material.

Supplementary file1 (DOCX 36 KB)

## Data Availability

All data generated or analyzed during this study are included in this published article (and its supplementary information file).

## References

[CR1] Ahmadi B, Lau CP, Giffin J, Santos N, Hahnel A, Raeside J, Christie H, Bartlewski P (2012). Suitability of epididymal and testicular ultrasonography and computerized image analysis for assessment of current and future semen quality in the ram. Exp Biol Med (Maywood).

[CR2] Aitken RJ (1999). The Amoroso Lecture. The human spermatozoon–a cell in crisis?. J Reprod Fertil.

[CR3] Alessi P, Ebbinghaus C, Neri D (2004). Molecular targeting of angiogenesis. Biochim Biophys Acta.

[CR4] Alexandre-Pires G, Mateus L, Martins C, Ferreira-Dias G (2012). Seasonal changes in testes vascularisation in the domestic cat (Felis domesticus): evaluation of microvasculature, angiogenic activity, and endothelial cell expression. Anat Res Int.

[CR5] Alonge S, Melandri M, Fanciullo L, Lacalandra GM, Aiudi G (2018). Prostate vascular flow: The effect of the ejaculation on the power doppler ultrasonographic examination. Reprod Domest Anim.

[CR6] Alonge S, Melandri M, Leoci R, Lacalandra GM, Aiudi G (2018). Ejaculation effect on blood testosterone and prostatic pulsed-wave Doppler ultrasound in dogs. Reprod Domest Anim.

[CR7] Araujo RR, Ginther OJ (2009). Vascular perfusion of reproductive organs in pony mares and heifers during sedation with detomidine or xylazine. Am J Vet Res.

[CR8] Ax RL, Dally M, Didion BA, Lenz RW, Love CC, Varner DD, Hafez B, Bellin ME, Hafez B, Hafez ESE (2000). Semen evaluation. Reproduction in farm animals.

[CR9] Barca Junior FA, Junior CK, Fávaro PC, Pereira GR, Menegassi SRO, Morotti F, Galdioli FHG, Souza AK, Barcellos JOJ, Seneda MM (2020). Infrared thermography and Doppler ultrasonography to evaluate the effects of scrotal insulation on testicular blood flow dynamics in bulls. Semina: Ciências Agrárias.

[CR10] Barclay AE (1947). Micro-arteriography. Br J Radiol.

[CR11] Barros Adwell CMQ, Brito LFC, Oba E, Wilde RE, Rizzoto G, Thundathil JC, Kastelic JP (2018). Arterial blood flow is the main source of testicular heat in bulls and higher ambient temperatures significantly increase testicular blood flow. Theriogenology.

[CR12] Barth AD, Waldner CL (2002). Factors affecting breeding soundness classification of beef bulls examined at the Western College of Veterinary Medicine. Can Vet J.

[CR13] Batissaco L, Celeghini ECC, Pinaffi FLV, de Oliveira BMM, de Andrade AFC, Recalde ECS, Fernandes CB (2013). Correlations between testicular hemodynamic and sperm characteristics in rams. Braz J Vet Res Anim Sci.

[CR14] Bergh A, Damber JE, de Kretser DM (1993). Vascular controls in testicular physiology. Molecular biology of the male reproductive system.

[CR15] Bergh A, Collin O, Lissbrant E (2001). Effects of acute graded reductions in testicular blood flow on testicular morphology in the adult rat. Biol Reprod.

[CR16] Bigliardi E, Denti L, De Cesaris V, Bertocchi M, Di Ianni F, Parmigiani E, Bresciani C, Cantoni AM (2019). Colour Doppler ultrasound imaging of blood flows variations in neoplastic and non-neoplastic testicular lesions in dogs. Reprod Domest Anim.

[CR17] Blanco PG, Arias DO, Gobello C (2008). Doppler ultrasound in canine pregnancy. J Ultrasound Med.

[CR18] Blazquez NB, Mallard GJ, Wedd SR (1988). Sweat glands of the scrotum of the bull. J Reprod Fertil.

[CR19] Blottner S, Wagener A, Schön J, Göritz F, Fickel J (2006). Reproductive fitness in roe bucks (Capreolus capreolus): Seasonal timing of testis function. Eur J Wildl Res.

[CR20] Bollwein H, Schulze JJ, Miyamoto A, Sieme H (2008). Testicular blood flow and plasma concentrations of testosterone and total estrogen in the stallion after the administration of human chorionic gonadotropin. J Reprod Dev.

[CR21] Bollwein H, Heppelmann M, Lüttgenau J (2016). Ultrasonographic Doppler use for female reproduction management. Vet Clin North Am Food Anim Pract.

[CR22] Boon JA, Boon JA (2011). The M-mode and doppler examination. Veterinary echocardiography.

[CR23] Boon JA, Boon JA (2011). Evaluation of size, function, and hemodynamics. Veterinary echocardiography.

[CR24] Boyd A, Pozor MA, Bailey CS, Verstegen J (2006). Effect of seasonality on testicular blood flow in mature stallions (Abstract). Anim Reprod Sci.

[CR25] Brito LF, Silva AE, Barbosa RT, Unanian MM, Kastelic JP (2003). Effects of scrotal insulation on sperm production, semen quality, and testicular echotexture in Bos indicus and Bos indicus x Bos taurus bulls. Anim Reprod Sci.

[CR26] Brito LF, Silva AE, Barbosa RT, Kastelic JP (2004). Testicular thermoregulation in Bos indicus, crossbred and Bos taurus bulls: relationship with scrotal, testicular vascular cone and testicular morphology, and effects on semen quality and sperm production. Theriogenology.

[CR27] Brito M, Feliciano M, Coutinho LN, Uscategui RR, Simões A, Maronezi MC, de Almeida VT, Crivelaro RM, Gasser B, Pavan L, Russiano WR (2015). Doppler and contrast-enhanced ultrasonography of testicles in adult domestic felines. Reprod Domest Anim.

[CR28] Brito MB, Maronezi MC, Uscategui RAR, Avante ML, Simoes AR, Monteiro FOB, Feliciano MAR (2018). Ultrasonographic methods for evaluation of testicles in cats Rev. MVZ Cordoba.

[CR29] Caires KC, de Avila J, McLean DJ (2009). (2009) Vascular endothelial growth factor regulates germ cell survival during establishment of spermatogenesis in the bovine testis. Reproduction.

[CR30] Camela ESC, Nociti RP, Santos VJC, Macente BI, Maciel GS, Feliciano MAR, Vicente WRR, Gill I, Bartlewski PM, Oliveira MEF (2017). Ultrasonographic characteristics of accessory sex glands and spectral Doppler indices of the internal iliac arteries in peri- and post-pubertal Dorper rams raised in a subtropical climate. Anim Reprod Sci.

[CR31] Camela ESC, Nociti RP, Santos VJC, Macente BI, Murawski M, Vicente WRR, Bartlewski PM, Oliveira MEF (2019). Changes in testicular size, echotexture, and arterial blood flow associated with the attainment of puberty in Dorper rams raised in a subtropical climate. Reprod Domest Anim.

[CR32] Carrillo JD, Soler M, Lucas X, Agut A (2012). Colour and pulsed Doppler ultrasonographic study of the canine testis. Reprod Domest Anim.

[CR33] Carvalho FA, Lammoglia MA, Simoes MJ, Randel RD (1995). Breed affects thermoregulation and epithelial morphology in imported and native cattle subjected to heat stress. J Anim Sci.

[CR34] Carvalho M, Mateus L, Afonso F, Van Harten S, Cardoso LA, Redmer DA, Ferreira-Dias G (2009). Testicular angiogenic activity in response to food restriction in rabbits. Reproduction.

[CR35] Cheng CY, Mruk DD (2012). The blood-testis barrier and its implications for male contraception. Pharmacol Rev.

[CR36] Claus LAM, Junior FAB, Junior CK, Pereira GR, Fávaro PC, Galdioli VHG, Seneda MM, Ribeiroa ELA (2019). Scrotal skin thickness, testicular shape and vascular perfusion using Doppler ultrasonography in bulls. Livest Sci.

[CR37] Colli LG, Belardin LB, Echem C, Akamine EH, Antoniassi MP, Andretta RR, Mathias LS, Rodrigues SFP, Bertolla RP, de Carvalho MHC (2019). Systemic arterial hypertension leads to decreased semen quality and alterations in the testicular microcirculation in rats. Sci Rep.

[CR38] Collin B (1973). La vascularisation artérielle du testicule chez le cheval. Anat Histol Embryol.

[CR39] Conway EM, Collen D, Carmeliet P (2001). Molecular mechanisms of blood vessel growth. Cardiovasc Res.

[CR40] de Souza MB, da Cunha BC, Pereira BS, Monteiro CL, Pinto JN, Linhares JC, da Silva LD (2014). Doppler velocimetric parameters of the testicular artery in healthy dogs. Res Vet Sci.

[CR41] de Souza MB, Barbosa CC, England GC, Mota Filho AC, Sousa CV, de Carvalho GG, Silva HV, Pinto JN, Linhares JC, Silva LD (2015). Regional differences of testicular artery blood flow in post pubertal and pre-pubertal dogs. BMC Vet Res.

[CR42] de Souza MB, England GC, Mota Filho AC, Ackermann CL, Sousa CV, de Carvalho GG, Silva HV, Pinto JN, Linhares JC, Oba E, da Silva LD (2015). Semen quality, testicular B-mode and Doppler ultrasound, and serum testosterone concentrations in dogs with established infertility. Theriogenology.

[CR43] de Souza Cunha DM, Barbosa de Souza M, Brito BF, Torres VL, Nunes TGP, Tavares SS, de Araujo VD, Machado da Silva LD, Pereira LMC, Teixeira DIA (2019). Testicular morphological and ultrasonographic characterization of male gray Brocket deers (Mazama gouazoubira) in different reproductive status. Acta Sci Vet.

[CR44] Dickey RP (1997). Doppler ultrasound investigation of uterine and ovarian blood flow in infertility and early pregnancy. Hum Reprod Update.

[CR45] Douglas RH, Umphenour N (1992). Endocrine abnormalities and hormonal therapy. Vet Clin North Am Equine Pract.

[CR46] Dudea SM, Ciurea A, Chiorean A, Botar-Jid C (2010). Doppler applications in testicular and scrotal disease. Med Ultrason.

[CR47] Dvorak HF, Nagy JA, Feng D, Brown LF, Dvorak AM (1999). Vascular permeability factor/vascular endothelial growth factor and the significance of microvascular hyperpermeability in angiogenesis. Curr Top Microbiol Immunol.

[CR48] Edwards JF (2008). Pathologic conditions of the stallion reproductive tract. Anim Reprod Sci.

[CR49] Elayat MA, Khalil KM, Farag FM, Rizk HM (2014). Gross anatomical studies on the pattern and density of the tunica vasculosa testis in some farm animals (buffalo, ram, camel, donkey and rabbit). BVMJ.

[CR50] Elbaz HT, Elweza AE, Sharshar AM (2019). Testicular color Doppler ultrasonography in Barki Rams. AJVS.

[CR51] Elmi A, Zannoni A, Govoni N, Bertocchi M, Forni M, Ventrella D, Bacci ML (2020). Uncovering the physiological mechanisms underlying the Roe Deer (Capreolus capreolus) testicular cycle: analyses of Gelatinases and VEGF patterns and correlation with testes weight and testosterone. Animals (Basel).

[CR52] El-Sherbiny HR, Abdelnaby EA, El-Shahat KH, Salem NY, Ramadan ES, Yehia SG, Fathi M (2022). Coenzyme Q10 Supplementation enhances testicular volume and hemodynamics, reproductive hormones, sperm quality, and seminal antioxidant capacity in goat bucks under summer hot humid conditions. Vet Res Commun.

[CR53] El-Sherbiny HR, Samir H, El-Shalofy AS, Abdelnaby EA (2022). Exogenous L-arginine administration improves uterine vascular perfusion, uteroplacental thickness, steroid concentrations and nitric oxide levels in pregnant buffaloes under subtropical conditions. Reprod Domest Anim.

[CR54] El-Sherbiny HR, El-Shalofy AS, Samir H (2022). Exogenous L-carnitine administration ameliorates the adverse effects of heat stress on testicular hemodynamics, echotexture, and total antioxidant capacity in rams. Front Vet Sci.

[CR55] El-Sherbiny HR, Fathi M, Samir H, Abdelnaby EA (2022). Supplemental dietary curcumin improves testicular hemodynamics, testosterone levels, and semen quality in Baladi bucks in the non-breeding season. Theriogenology.

[CR56] El-Sherbiny H, Shahat A, Hedia M, El-Shalofy A (2022). Effect of sexual maturation on testicular morphometry and echotexture and their association with intratesticular blood flow in ossimi rams. Indian J Small Rumin.

[CR57] England G, Bright L, Pritchard B, Bowen IM, de Souza MB, Silva L, Moxon R (2017). Canine reproductive ultrasound examination for predicting future sperm quality. Reprod Domest Anim.

[CR58] Fadl AM, Abdelnaby EA, El-Sherbiny HR (2022). Supplemental dietary zinc sulphate and folic acid combination improves testicular volume and haemodynamics, testosterone levels and semen quality in rams under heat stress conditions. Reprod Domest Anim.

[CR59] Fávaro PPC, Pereira GR, Junior FAB, Adona PR, Franco EMV, Dias IDS, Seneda MM, Junior CK (2020). Hemodynamic evaluation of the supratesticular artery in bulls. Livest Sci.

[CR60] Fowler D, Setchell B (1971). Selecting Merino rams for ability to withstand infertility caused by heat. 2. The effect of heat on scrotal and testicular blood flow. Austral J Exper Agric.

[CR61] Gacem S, Papas M, Catalan J, Miró J (2020). Examination of jackass (Equus asinus) accessory sex glands by B-mode ultrasound and of testicular artery blood flow by colour pulsed-wave Doppler ultrasound: Correlations with semen production. Reprod Domest Anim.

[CR62] Ginther OJ (2007). Ultrasonic Imaging and Animal Reproduction (Ed.): Color- Doppler Ultrasonography.

[CR63] Ginther OJ, Utt MD (2004). Doppler ultrasound in equine reproduction: principles, techniques, and potential. J Equine Vet Sci.

[CR64] Glode LM, Robinson J, Horwitz LD (1984). Scrotal hypothermia and testicular blood flow in the dog. Absence of thermal regulation. J Androl.

[CR65] Gloria A, Carluccio A, Wegher L, Robbe D, Valorz C, Contri A (2018). Pulse wave Doppler ultrasound of testicular arteries and their relationship with semen characteristics in healthy bulls. J Anim Sci Biotechnol.

[CR66] Gloria A, Di Francesco L, Marruchella G, Robbe D, Contri A (2020). Pulse-wave Doppler pulsatility and resistive indexes of the testicular artery increase in canine testis with abnormal spermatogenesis. Theriogenology.

[CR67] Gloria A, Candeloro L, Wegher L, Robbe D, Carluccio A, Contri A (2021). Environmental temperature and relative humidity differently affect the sperm characteristics in Brown Swiss and Belgian Blue bulls. Int J Biometeorol.

[CR68] Gonzalvo V, Calvo MA, Navalon P, Cejalvo D, Ramada FJ, Blasco JE, Donderis C, Lloris JM (1993). Role of testosterone in the testicular microcirculatory changes produced in the rat by the administration of high doses of human chorionic gonadotrophin. Arch Esp Urol.

[CR69] Gouletsou PG (2017). Ultrasonographic examination of the scrotal contents in rams. Small Rumin Res.

[CR70] Gouletsou PG, Amiridis GS, Cripps PJ, Lainas T, Deligiannis K, Saratsis P, Fthenakis GC (2003). Ultrasonographic appearance of clinically healthy testicles and epididymides of rams. Theriogenology.

[CR71] Gumbsch P, Gabler C, Holzmann A (2002). Colour-coded duplex sonography of the testes of dogs. Vet Rec.

[CR72] Günzel-Apel AR, Möhrke C, Poulsen Nautrup C (2001). Colour-coded and pulsed Doppler sonography of the canine testis, epididymis and prostate gland: physiological and pathological findings. Reprod Domest Anim.

[CR73] Hamilton TR, Mendes CM, de Castro LS, de Assis PM, Siqueira AF, Delgado Jde C, Goissis MD, Muiño-Blanco T, Cebrián-Pérez JÁ, Nichi M, Visintin JA, Assumpção ME (2016). Evaluation of lasting effects of heat stress on sperm profile and oxidative status of ram semen and Epididymal sperm. Oxid Med Cell Longev.

[CR74] Hansen PJ (2009). Effects of heat stress on mammalian reproduction. Philos Trans R Soc Lond B Biol Sci.

[CR75] Hao WY, Shao CH, Feng YL, Hu JT, Li Q, Wang HQ, Wang PT (2013). Hypoxia reduces the proliferation and occludin expression of primary sertoli cells. Zhonghua Nan Ke Xue.

[CR76] Harrison RG (1949). The comparative anatomy of the blood supply of the mammalian testis. Proc Zool Soc London.

[CR77] Harrison RG, Weiner JS (1949). Vascular patterns of the mammalian testis and their functional significance. J Exp Biol.

[CR78] Hassan MAA, Sayed RKA, Abdelsabour-Khalaf M, Abd-Elhafez EA, Anel-Lopez L, Riesco MF, Ortega-Ferrusola C, Montes-Garrido R, Neila-Montero M, Anel L, Alvarez M (2022). Morphological and ultrasonographic characterization of the three zones of supratesticular region of testicular artery in Assaf rams. Sci Rep.

[CR79] Hedia MG, El-Belely MS (2021). Testicular morphometric and echotextural parameters and their correlation with intratesticular blood flow in Ossimi ram lambs. Large Anim Rev.

[CR80] Hedia M, El-Shalofy A (2022). Ageing affects plasma steroid concentrations and testicular volume, echotexture and haemodynamics in rams. Andrologia.

[CR81] Hedia MG, El-Belely MS, Ismail ST, Abo El-Maaty AM (2019). Monthly changes in testicular blood flow dynamics and their association with testicular volume, plasma steroid hormones profile and semen characteristics in rams. Theriogenology.

[CR82] Hedia MG, El-Belely MS, Ismail ST, Abo El-Maaty AM (2020). Seasonal variation in testicular blood flow dynamics and their relation to systemic and testicular oxidant/antioxidant biomarkers and androgens in rams. Reprod Domest Anim.

[CR83] Hedia MG, El-Belely MS, Ismail ST, Abo El-Maaty AM (2020). Evaluation of testicular blood flow and ultrasonographic measurements in rams with emphasis on laterality. J Adv Vet Res.

[CR84] Henning H, Masal C, Herr A, Wolf K, Urhausen C, Beineke A, Beyerbach M, Kramer S, Günzel-Apel AR (2014). Effect of short-term scrotal hyperthermia on spermatological parameters, testicular blood flow and gonadal tissue in dogs. Reprod Domest Anim.

[CR85] Herzog K, Bollwein H (2007). Application of Doppler ultrasonography in cattle reproduction. Reprod Domest Anim.

[CR86] Hsu HS, Chang LS, Chen MT, Wei YH (1994). Decreased blood flow and defective energy metabolism in the varicocele-bearing testicles of rats. Eur Urol.

[CR87] Jantosovicová J, Jantosovic J (1983). Topographico-anatomic data on the testicular artery, ductus deferens artery and cremaster artery in the stallion. Gegenbaurs Morphol Jahrb.

[CR88] Joffre J, Joffre M (1973). Seasonal changes in the testicular blood flow of seasonally breeding mammals: dormouse, Glis glis, ferret, Mustella furo, and fox, Vulpes Vulpes. J Reprod Fertil.

[CR89] Johnston JE, Naelapaa H, Frye JB (1963). Physiological responses of Holstein. Brown Swiss and Red Sindhi crossbreed bulls exposed to high temperatures and humidities. J Anim Sci.

[CR90] Junior FAB, Junior CK, Fávaro PDC, Pereira GR, Morotti F, Menegassi SRO, Barcellos JOJ, Seneda MM (2018). Effect of breed on testicular blood flow dynamics in bulls. Theriogenology.

[CR91] Junior FAB, Junior CK, Fávaro PC, Pereira GR, Menegassi SRO, Morotti F, Galdioli FHG, Souza AK, Barcellos JOJ, Seneda MM (2020). Infrared thermography and Doppler ultrasonography to evaluate the effects of scrotal insulation on testicular blood flow dynamics in bulls. Semina: Ciências Agrárias, Londrina.

[CR92] Kastelic JP, Cook RB, Coulter GH, Saacke RG (1996). Insulating the scrotal neck affects semen quality and scrotal/testicular temperatures in the bull. Theriogenology.

[CR93] Kastelic JP, Cook RB, Coulter GH (1997). Contribution of the scrotum, testes, and testicular artery to scrotal/testicular thermoregulation in bulls at two ambient temperatures. Anim Reprod Sci.

[CR94] Kastelic JP, Wilde RE, Rizzoto G, Thundathil JC (2017). Hyperthermia and not hypoxia may reduce sperm motility and morphology following testicular hyperthermia. Vet Med.

[CR95] Kastelic JP, Rizzoto G (2021) Thermoregulation of the Testes. Bovine Reproduction, pp:40–46. 10.1002/9781119602484.ch4

[CR96] Kay GW, Grobbelaar JA, Hattingh J (1992). Effect of surgical restriction of growth of the testicular artery on testis size and histology in bulls. J Reprod Fertil.

[CR97] Khadamy J, Abri Aghdam K, Falavarjani KG (2018). An update on optical coherence tomography angiography in diabetic retinopathy. J Ophthalmic vis Res.

[CR98] Khalil KM (2014) Gross anatomical studies on the pattern and density of the tunica vasculosa testis in some farm animals (buffalo, ram, camel, donkey and rabbit). Master thesis, Cairo University, Egypt, 2014. https://scholar.cu.edu.eg/karimkhalil/files/31_october_2013.pdf

[CR99] Kozłowska N, Faundez R, Borzyszkowski K, Dąbrowski S, Jasiński T, Domino M (2022). The relationship between the testicular blood flow and the semen parameters of rams during the selected periods of the breeding and non-breeding seasons. Animals (Basel).

[CR100] Kutzler M, Tyson R, Grimes M, Timm K (2011). Determination of testicular blood flow in camelids using vascular casting and color pulsed-wave Doppler ultrasonography. Vet Med Int.

[CR101] LeCouter J, Ferrara N (2002). EG-VEGF and the concept of tissue-specific angiogenic growth factors. Semin Cell Dev Biol.

[CR102] Lemos H, Dorado J, Hidalgo M, Gaivão I, Martins-Bessa A (2020). Assessment of dog testis perfusion by colour and pulsed-doppler ultrasonography and correlation with sperm oxidative DNA damage. Top Companion Anim Med.

[CR103] Liu X, Yang Y (2004). Effect of VEGF on the angiogenesis in male reproduction system. Zhonghua Nan Ke Xue.

[CR104] Llamas-Luceño N, Hostens M, Mullaart E, Broekhuijse M, Lonergan P, Van Soom A (2020). High temperature-humidity index compromises sperm quality and fertility of Holstein bulls in temperate climates. J Dairy Sci.

[CR105] Lloyd-Jones JL, Purohit RC, Boyle M, Shepherd C (2015). Use of thermography for functional evaluation of stallion scrotum and testes. J Equine Vet Sci.

[CR106] Mandour AS, Samir H, El-Beltagy MA, Abdel-Daim MM, Izumi W, Ma D, Matsuura K, Tanaka R, Watanabe G (2020). Effect of supra-nutritional selenium-enriched probiotics on hematobiochemical, hormonal, and Doppler hemodynamic changes in male goats. Environ Sci Pollut Res Int.

[CR107] Mariotti A, Di Carlo L, Orlando G, Corradini ML, Di Donato L, Pompa P, Iezzi R, Cotroneo AR, Romani GL, Merla A (2011). Scrotal thermoregulatory model and assessment of the impairment of scrotal temperature control in varicocele. Ann Biomed Eng.

[CR108] Markey CM, Jequier AM, Meyer GT, Martin GB (1995). Relationship between testicular morphology and sperm production following ischaemia in the ram. Reprod Fertil Dev.

[CR109] Max B (1992). This and that: hair pigments, the hypoxic basis of life and the Virgilian journey of the spermatozoon. Trends Pharmacol Sci.

[CR110] Mayerhofer A, Sinha Hikim AP, Bartke A, Russell LD (1989). Changes in the testicular microvasculature during photoperiod-related seasonal transition from reproductive quiescence to reproductive activity in the adult golden hamster. Anat Rec.

[CR111] Middleton WD, Thorne DA, Melson GL (1989). Color Doppler ultrasound of the normal testis. AJR Am J Roentgenol.

[CR112] Mieusset R, Sowerbutts SF, Zupp JL, Setchell BP (1992). Increased flow of testicular blood plasma during local heating of the testes of rams. J Reprod Fertil.

[CR113] Morrell JM (2020). Heat stress and bull fertility. Theriogenology.

[CR114] Nichi M, Bols PE, Züge RM, Barnabe VH, Goovaerts IG, Barnabe RC, Cortada CN (2006). Seasonal variation in semen quality in Bos indicus and Bos taurus bulls raised under tropical conditions. Theriogenology.

[CR115] Nolte T, Harleman JH, Jahn W (1995). Histopathology of chemically induced testicular atrophy in rats. Exp Toxicol Pathol.

[CR116] Noordhuizen-Stassen EN, Charbon GA, de Jong FH, Wensing CJ (1985). Functional arterio-venous anastomoses between the testicular artery and the pampiniform plexus in the spermatic cord of rams. J Reprod Fertil.

[CR117] Ntemka A, Kiossis E, Boscos C, Theodoridis A, Kourousekos G, Tsakmakidis I (2018). Effects of testicular hemodynamic and echogenicity changes on ram semen characteristics. Reprod Domest Anim.

[CR118] Ortega-Ferrusola C, Gracia-Calvo LA, Ezquerra J, Pena FJ (2014). Use of colour and spectral Doppler ultrasonography in stallion andrology. Reprod Domest Anim.

[CR119] Ortiz-Rodriguez JM, Anel-Lopez L, Martín-Muñoz P, Álvarez M, Gaitskell-Phillips G, Anel L, Rodríguez-Medina P, Peña FJ, Ortega Ferrusola C (2017). Pulse Doppler ultrasound as a tool for the diagnosis of chronic testicular dysfunction in stallions. PLoS ONE.

[CR120] Oyen RH (2002). Scrotal ultrasound. Eur Radiol.

[CR121] Paltiel HJ, Rupich RC, Babcock DS (1994). Maturational changes in arterial impedance of the normal testis in boys: Doppler sonographic study. AJR Am J Roentgenol.

[CR122] Paul C, Murray AA, Spears N, Saunders PT (2008). A single, mild, transient scrotal heat stress causes DNA damage, subfertility and impairs formation of blastocysts in mice. Reproduction.

[CR123] Paul C, Teng S, Saunders PT (2009). A single, mild, transient scrotal heat stress causes hypoxia and oxidative stress in mouse testes, which induces germ cell death. Biol Reprod.

[CR124] Pérez B, Mateos E (1996). Effect of photoperiod on semen production and quality in bucks of Verata and Malaguen ~a breeds. Small Rum Res.

[CR125] Pinggera GM, Mitterberger M, Bartsch G, Strasser H, Gradl J, Aigner F, Pallwein L, Frauscher F (2008). Assessment of the intratesticular resistive index by colour Doppler ultrasonography measurements as a predictor of spermatogenesis. BJU Int.

[CR126] Polguj M, Wysiadecki G, Podgórski M, Szymański J, Olbrych K, Olewnik Ł, Topol M (2015). Morphological variations of intra-testicular arterial vasculature in bovine testis–a corrosion casting study. BMC Vet Res.

[CR127] Pozor MA (2007). Evaluation of testicular vasculature in stallions. Clin Tech Equine Pract.

[CR128] Pozor MA, McDonnell SM (2004). Color Doppler ultrasound evaluation of testicular blood flow in stallions. Theriogenology.

[CR129] Pozor MA, Muehlhaus J, King A, Macpherson ML, Troedsson MH, Bailey CS (2011). Effect of pentoxifylline treatment on testicular perfusion and semen quality in Miniature horse stallions. Theriogenology.

[CR130] Pozor MA, Nolin M, Roser J, Runyon S, Macperson ML, Kelleman A (2014). Doppler indices of vascular impedance as indicators of testicular dysfunction in stallions. J Equine Vet Sci.

[CR131] Pugliesi G, Miagawa BT, Paiva YN, França MR, Silva LA, Binelli M (2014). Conceptus-induced changes in the gene expression of blood immune cells and the ultrasound-accessed luteal function in beef cattle: how early can we detect pregnancy?. Biol Reprod.

[CR132] Pugliesi G, Dalmaso de Melo G, Silva JB, Carvalhêdo AS, Lopes E, de Siqueira FE, Silva LA, Binelli M (2019). Use of color-Doppler ultrasonography for selection of recipients in timed-embryo transfer programs in beef cattle. Theriogenology.

[CR133] Pyter LM, Hotchkiss AK, Nelson RJ (2005). Photoperiod-induced differential expression of angiogenesis genes in testes of adult Peromyscus leucopus. Reproduction.

[CR134] Raisi A, Davoodi F (2022). Testicular torsions in veterinary medicine. Vet Res Commun.

[CR135] Reddy N, Kasukurthi KB, Mahla RS, Pawar RM, Goel S (2012). Expression of vascular endothelial growth factor (VEGF) transcript and protein in the testis of several vertebrates, including endangered species. Theriogenology.

[CR136] Rizzoto G, Kastelic JP (2020). A new paradigm regarding testicular thermoregulation in ruminants?. Theriogenology.

[CR137] Rizzoto G, Ferreira JCP, Mogollón Garcia HD, Teixeira-Neto FJ, Bardella LC, Martins CL, Silva JRB, Thundathil JC, Kastelic JP (2020). Short-term testicular warming under anesthesia causes similar increases in testicular blood flow in Bos taurus versus Bos indicus bulls, but no apparent hypoxia. Theriogenology.

[CR138] Rodrigues NN, Rossi GF, Vrisman DP, Taira AR, Souza LL, Zorzetto MF, Bastos NM, de Paz CCP, de Lima VFMH, Monteiro FM, Franco Oliveira ME (2020). Ultrasonographic characteristics of the testes, epididymis and accessory sex glands and arterial spectral indices in peri- and post-pubertal Nelore and Caracu bulls. Anim Reprod Sci.

[CR139] Samir H, Kandiel MMM (2019). Accuracy of subjective evaluation of luteal blood flow by color Doppler ultrasonography for early diagnosis of pregnancy in Egyptian buffalo. Anim Reprod Sci.

[CR140] Samir H, Sasaki K, Ahmed E, Karen A, Nagaoka K, El Sayed M, Taya K, Watanabe G (2015). Effect of a single injection of gonadotropin-releasing hormone (GnRH) and human chorionic gonadotropin (hCG) on testicular blood flow measured by color doppler ultrasonography in male Shiba goats. J Vet Med Sci.

[CR141] Samir H, Nyametease P, Nagaoka K, Watanabe G (2018). Effect of seasonality on testicular blood flow as determined by color Doppler ultrasonography and hormonal profiles in Shiba goats. Anim Reprod Sci.

[CR142] Samir H, Kandiel MMM, El-Maaty AMA, Sediqyar M, Sasaki K, Watanabe G (2019). Ovarian follicular changes and hemodynamics in Egyptian buffaloes under CIDR-PGF2α and Ovsynch-CIDR estrus synchronization treatments. J Reprod Dev.

[CR143] Samir H, Nyametease P, Elbadawy M, Nagaoka K, Sasaki K, Watanabe G (2020). Administration of melatonin improves testicular blood flow, circulating hormones, and semen quality in Shiba goats. Theriogenology.

[CR144] Samir H, El Sayed MAI, Nagaoka K, Sasaki K, Abo El-Maaty AM, Karen A, Abou-Ahmed MM, Watanabe G (2020). Passive immunization against inhibin increases testicular blood flow in male goats. Theriogenology.

[CR145] Samir H, Radwan F, Watanabe G (2021). Advances in applications of color Doppler ultrasonography in the andrological assessment of domestic animals: A review. Theriogenology.

[CR146] Samir H, El-Shalofy AS, El-Sherbiny HR (2023) Effects of a single dose of long-acting FSH on testicular blood flow, testicular echotexture, and circulating testosterone, estradiol, and nitric oxide in rams during the non-breeding season. Domest Anim Endocrinol 82:106765. 10.1016/j.domaniend.2022.106765 10.1016/j.domaniend.2022.106765 36219897

[CR147] Samir H, Mandour AS, Radwan F, Swelum AA, Yoshida T, Tanaka R, Nagaoka K, Watanabe G (2022). Diurnal rhythms in testicular blood flow, testicular morphometry and reproductive hormones in Shiba goats. Reprod Fertil Dev.

[CR148] Samper JC, Pycock JF, McKinnon AO (2007). Current therapy in equine reproduction.

[CR149] Sargent KM, Clopton DT, Lu N, Pohlmeier WE, Cupp AS (2016). VEGFA splicing: divergent isoforms regulate spermatogonial stem cell maintenance. Cell Tissue Res.

[CR150] Saunders HM, Burns PN, Needleman L, Liu JB, Boston R, Wortman JA, Chan L (1998). Hemodynamic factors affecting uterine artery Doppler waveform pulsatility in sheep. J Ultrasound Med.

[CR151] Serin G, Gökdal O, Tarimcilar T, Atay O (2010). Umbilical artery doppler sonography in Saanen goat fetuses during singleton and multiple pregnancies. Theriogenology.

[CR152] Setchell BP, Setchell BP (1978). The scrotum and thermoregulation. The mammalian testis.

[CR153] Setchell BP (1998). Heat and the testis. J Reprod Fertil.

[CR154] Setchell BP (2006) The effects of heat on the testes of mammals. J Reprod Fertil 114:179–194. Anim Reprod 3(2):81–91. https://www.animal-reproduction.org/article/5b5a607ef7783717068b47c0

[CR155] Setchell BP, Waites GM, Thorburn GD (1966). Blood flow in the testis of the conscious ram measured with krypton85. Circ Res.

[CR156] Setchell BP, Maddocks S, Brooks DE, Knobil E, Neill JD (1994). Anatomy, vasculature, innervation, and fluids of the male reproductive tract. The physiology of reproduction.

[CR157] Shahat AM, Rizzoto G, Kastelic JP (2020). Amelioration of heat stress-induced damage to testes and sperm quality. Theriogenology.

[CR158] Siqueira JB, Oba E, Pinho RO, Guimarães SEF, Miranda Neto T, Guimarães JD (2012). Testicular shape and andrological aspects of young Nellore bulls underextensive farming. R Bras Zootec.

[CR159] Skinner JD, Louw GN (1966). Heat stress and spermatogenesis in Bos indicus and Bos taurus cattle. J Appl Physiol.

[CR160] Souza MB, Mota Filho AC, Sousa CVS, Monteiro CLB, Carvalho GG, Pinto JN, Jussiara CS, Linhares JCS, Silva LDM (2014). Triplex Doppler evaluation of the testes in dogs of different sizes. Pesqui Vet Bras.

[CR161] Strina A, Corda A, Nieddu S, Solinas G, Lilliu M, Zedda M, Pau S, Ledda S (2016). Annual variations in resistive index (RI) of testicular artery, volume measurements and testosterone levels in bucks. Comp Clin Path.

[CR162] Sweeney TE, Rozum JS, Desjardins C, Gore RW (1991). Microvascular pressure distribution in the hamster testis. Am J Physiol.

[CR163] Trautwein LGC, Souza AK, Martins MIM (2019). Can testicular artery Doppler velocimetry values change according to the measured region in dogs?. Reprod Domest Anim.

[CR164] Viana JH, Arashiro EK, Siqueira LG, Ghetti AM, Areas VS, Guimarães CR, Palhao MP, Camargo LS, Fernandes CA (2013). Doppler ultrasonography as a tool for ovarian management. Anim Reprod.

[CR165] Vogler CJ, Saacke RG, Bame JH, Dejarnette JM, McGilliard ML (1991). Effects of scrotal insulation on viability characteristics of cryopreserved bovine semen. J Dairy Sci.

[CR166] Wagener A, Blottner S, Göritz F, Streich WJ, Fickel J (2003). Differential changes in expression of a and b FGF, IGF-1 and -2, and TGF-alpha during seasonal growth and involution of roe deer testis. Growth Factors.

[CR167] Wagener A, Fickel J, Schön J, Fritzenkötter A, Göritz F, Blottner S (2005). Seasonal variation in expression and localization of testicular transforming growth factors TGF-{beta}1 and TGF-{beta}3 corresponds with spermatogenic activity in roe deer. J Endocrinol.

[CR168] Wagener A, Blottner S, Göritz F, Streich WJ, Fickel J (2010). Circannual changes in the expression of vascular endothelial growth factor in the testis of roe deer (Capreolus capreolus). Anim Reprod Sci.

[CR169] Waites GMH, Johnson AD, Gomes WR, Vandemark NL (1970). Temperature regulation and the testis. Development, anatomy, and physiology.

[CR170] Waites GM, Setchell BP, Quinlan D (1973). Effect of local heating of the scrotum, testes and epididymides of rats on cardiac output and regional blood flow. J Reprod Fertil.

[CR171] Widmark A, Damber JE, Bergh A (1986). Relationship between human chorionic gonadotrophin-induced changes in testicular microcirculation and the formation of testicular interstitial fluid. J Endocrinol.

[CR172] Zagzebski JA (2005). Physics and instrumentation in Doppler and B-Mode ultrasonography.

[CR173] Zaidi J, Jurkovic D, Campbell S, Okokon E, Tan SL (1995). Circadian variation in uterine artery blood flow indices during the follicular phase of the menstrual cycle. Ultrasound Obstet Gynecol.

[CR174] Zelli R, Troisi A, Elad Ngonput A, Cardinali L, Polisca A (2013). Evaluation of testicular artery blood flow by Doppler ultrasonography as a predictor of spermatogenesis in the dog. Res Vet Sci.

