# Supplementary material for: An updated insight on testicular hemodynamics: Environmental, physiological, and technical perspectives in farm and companion animals
Source: Vet Res Commun. 2022 Nov 18;47(2):323–45. doi: 10.1007/s11259-022-10022-9 (PMC10209317; doi:10.1007/s11259-022-10022-9)
Supplement: Supplementary file 1 — Supplementary file1 (DOCX 36 KB) [file 11259_2022_10022_MOESM1_ESM.docx]

**Table 1. Characterizations of testicular hemodynamics as measured by pulsed Doppler ultrasonography in different studies on bulls, rams, goats, dear, and alpaca**

| **Species** | **Breeds** | **Age** | **No** | **B. W** | **Season** | **Testicular hemodynamics at the STA** | | | | | | **Other portions of the testicular artery** | **References** |
| --- | --- | --- | --- | --- | --- | --- | --- | --- | --- | --- | --- | --- | --- |
|  |  |  |  |  |  | **PSV (cm/s)** | **EDV (cm/s)** | **RI** | **PI** | **TAMAX (cm/s)** | **MV (cm/s)** |  |  |
| **Bulls** | Various breeds (Angus, Braford, Brangus, Hereford, Nelore) | 18-36 months | 334 | NI | NI | NI | NI | 0.41 ± 0.01 | 0.33 ± 0.01 | NI | 12.14±0.30 | NI | Junior et al (2018) |
|  | Aberdeen Angus and Polled Hereford | 12-18 months | 28 | NI | All-over the year | NI | NI | 0.39±0.20 | 0.45±0.16 | NI | 11.43±3.65 | NI | Fávaro et al (2020) |
|  | Nelore and Caracu bulls | 13.70±0.09 months | 282 | 362.56±5.53 kg | NI | 16.83±3.94 | NI | 0.45±0.01 | 0.65±0.03 | NI | NI | NI | Rodrigues et al (2020) |
|  | Braford bulls | 16 months | 106 | NI | Breeding season (January) | 19.11±6.25 | 10.00±4.17 | 0.48±0.17 | 0.49±0.26 | NI | 14.32±4.85 | NI | Claus et al (2019) |
|  | Braford bulls (3 Nellore -5 Hereford) | 18 months | 8 | 531.28 ± 23.45 kg | October to December | NI | NI | 0.34 ± 0.04 | 0.25 ± 0.06 | NI | 13.09±0.19 | NI | Junior et al (2020) |
|  | Swiss Brown bulls | 4.29±1.30 years | 18 | NI | NI | 13.95±4.45 | 5.45±2.10 | 0.61±0.10 | NI | NI | NI | MTA: PSV:6.40±1.30, EDV: 4.25±0.95, RI:0.43±0.09; ITA: PSV:5.78±1.70, EDV: 3.80±0.85, RI:0.34±0.08 | Gloria et al (2018) |

**Cont. Table 1. Characterizations of testicular hemodynamics as measured by pulsed Doppler ultrasonography in different studies on bulls, rams, goats, dear, and alpaca**

| **Species** | **Breeds** | **Age** | **No** | **B. W** | **Season** | **Testicular hemodynamics at the STA** | | | | | | **Other portions of the testicular artery** | **References** |
| --- | --- | --- | --- | --- | --- | --- | --- | --- | --- | --- | --- | --- | --- |
|  |  |  |  |  |  | **PSV (cm/s)** | **EDV (cm/s)** | **RI** | **PI** | **TAMAX (cm/s)** | **MV (cm/s)** |  |  |
| **Rams** | Dorper rams | 12.80±0.60 months | 45 | 65.80±2.65 kg | NI | 17.40±0.85 | 6.00±0.65 | 0.64±0.04 | 1.3±0.2 | NI | NI | NI | Camela et al (2019) |
|  | Barki rams | 2-2.5 years | 12 | 50- 60 kg | Breeding season (Autumn) | NI | NI | 0.60±0.04 | 1.01±0.07 | NI | NI | MTA: RI:0.33±0.04, PI: 0.58±0.04 | Elweza et al (2021) |
|  | Awassi rams | 3-5 years | 5 | 50-65 kg | NI | NI | NI | 0.45±0.02 | 0.64±0.05 | NI | NI | NI | Hedia et al (2020b) |
|  | Fat-tailed rams | 2–4 years | 5 | 45–60 kg | All over the year | NI | NI | 0.3-0.5 | 0.4-0.9 | NI | NI | NI | Hedia et al (2019) |
| **Goats** | Sarda bucks | 28 months | 3 | NI | All over the year | NI | NI | 0.70-0.85 | NI | NI | NI | NI | Strina et al (2016) |
|  | Shiba goats | 22.5±3.5 months | 12 | 26.85 ± 3.25 kg | Spring | 15.51±0.38 | 6.47±0.53 | 0.456±0.030 | 0.635±0.058 | 10.08±0.62 | NI | MTA: PSV: 10.72±0.67, EDV: 7.54±0.45, 0.226±0.015, 0.260±0.032 | Samir et al (2020a) |
|  | Shiba goats | 17.5±1.5 months | 9 | 20.30 ± 2.17 kg | Winter | NI | NI | 0.4-0.6 | 0.5-0.8 | NI | NI | MTA: RI:0.3-0.45, PI:0.4-0.55 | Samir et al (2020b) |
|  | Shiba goats | 19.5±2.5 months | 12 | 24.30 ± 4.17 kg | All over the year | 15.89±0.49 | 8.01±0.24 | 0.49±0.01 | 0.69±0.02 | 11.56±0.34 | NI | NI | Samir et al (2018) |
|  | Shiba goats | 2 years | 5 | 25 ± 3 kg | Summer | 24.1 ± 2.03 | 7.4 ± 0.7 | 0.66 ± 0.0 | 1.1 ± 0.1 | NI | 14.2±0.9 | NI | Mandour et al (2020) |
| **Deer** | gray brocket deer (M. gouazoubira) | 5-15 years | 2 | 15.00±0.00 kg | All over the year | 10.66±5.60 | 5.59±2.17 | 0.40±0.10 | 0.89±0.75 | 6.38±2.62 | NI | NI | de Souza Cunha et al (2019) |
| **Alpaca** | alpaca | 3-13 months | 7 | 140.71±3.13 kg | NI | 21.41±1.11 | 6.51±0.81 | 0.69±0.04 | NI | NI | NI | MTA: PSV: 13.18±0.94, EDV:7.29±0.57, RI: 0.45±0.03 | Kutzler et al (2011) |

**Table 2. Characterizations of testicular hemodynamics as measured by pulsed Doppler ultrasonography in different studies on stallions and dogs**

| **Species** | **Breeds** | **Age** | **No** | **B. W** | **Season** | **Testicular hemodynamics in different portions of testicular arteries** | | | | | | **References** |
| --- | --- | --- | --- | --- | --- | --- | --- | --- | --- | --- | --- | --- |
|  |  |  |  |  |  | **PSV** | **EDV** | **RI** | **PI** | **TAMAX** | **MV and Others** |  |
| **Stallion** | Various breeds | 6-18 years | 7 | NI | NI | STA:24.96±6.58, MTA: 18.5±, ITA:10.08±2.59 | 4.85±1.34, MTA:6.5, ITA:4.26±1.14 | STA: 0.80±0.05, MTA:0.62, ITA:0.57±0.08 | STA: 2.28±0.45, MTA:1.5, ITA:0.90±0.21 | STA: 8.82±1.89, MTA:8.5, ITA: 6.50 ± 1.64 |  | Ortiz-Rodriguez et al (2017) |
|  | Miniature stallions | NI | 6 | NI | NI | NI | NI | MTA: 0.52±0.03 | MTA: 1.3±0.09 | NI | NI | Pozor et al (2014) |
|  | Various breeds | 3–22 years | 52 | NI | Breeding season (May and June) | STA: 26.1±0.91 (12–51), MTA: 22.2±1.22(8–59) | STA: 5.4±0.31(0.3–14.7), MTA:7.9±0.42(3.5–20.0) | STA: 0.78±0.01(0.56–0.99), MTA: 0.63±0.012(0.39–0.85) | STA: 1.99±0.076(0.96–3.96), MTA: 1.15±0.045(0.55–2.29) | NI | NI | Pozor and McDonnell (2004) |
|  | Hanoverian | 12.5±1.3 years | 12 | NI | July to August | NI | NI | 1.79±0.18 to 2.16±0.23 | NI | NI | BFV =27.4±6.6 to 44.1±11.7 | Bollwein et al (2008) |
|  | Various breeds | NI | 40 | NI | NI | NI | NI | 0.8±0.1 (0.5-1.0) | 2.2±0.6 (0.8-6.4) | 10.4±1.9 (5.1-17.6) | BFV=33.4±6.5 (14.9-80.4); TAD=2.6±0.2 (2-3.9) | Scheibenzuber (2005) |
|  | Miniature stallions | 3-8 years | 6 | NI | Breeding season (July) | MTA: 12 to 15 | MTA: 4 to 5 | MTA: 0.7-0.8 | MTA: 1.4-2 | MTA: 5.8-8.2 | MTA: BFV=12-23 | Pozor et al (2011) |

**Cont. Table 2. Characterizations of testicular hemodynamics as measured by pulsed Doppler ultrasonography in different studies on stallions and dogs**

| **Species** | **breeds** | | | **Age** | **No** | | | **B. W** | **Season** | **Testicular hemodynamics in different portions of testicular arteries** | | | | | | **References** |
| --- | --- | --- | --- | --- | --- | --- | --- | --- | --- | --- | --- | --- | --- | --- | --- | --- |
|  |  |  |  |  |  |  |  |  |  | **PSV** | **EDV** | **RI** | **PI** | **TAMAX** | **MV and Others** |  |
| **Dogs** | Various breeds | | | 3-5 years | 5 | | | 25-35 kg | NI | 13.13 ± 0.63 to 17.75 ± 1.08 | 6.02 ± 1.28 to 9.17 ± 2.18 | 0.47 ± 0.05 to 0.66 ± 1.12 | 0.64 ± 0.05 to 1.01 ± 0.10 |  | NI | Zelli et al (2013) |
|  | Various breeds | | | 2 -8 years old (4.5±1.9 years) | 10 | | | 33-42 kg | NI | STA: 11.35±2.61, MTA:11.22±3.00, ITA:6.83±0.99 | STA: 6.67±1.66, MTA:6.80±1.72, ITA:4.74±0.84 | STA: 0.49±0.12, MTA:0.38±0.10, ITA:0.31±0.07 | STA: 0.75±0.29, MTA: 0.49±0.14, ITA: 0.39±0.10 | NI | NI | de Souza et al (2015a) |
|  | Various breeds | | | 0.5-4 years | 20 | | | 15-42 kg | NI | STA: 10.66±2.23, MTA: 11.27±2.54, ITA: 6.15±1.00 | STA: 5.79±1.51, MTA: 6.21±1.70, ITA:4.28±0.79 | STA: 0.43±0.12, MTA:0.34±0.09, ITA: 0.30±0.05 | STA: 0.61±0.22, MTA: 0.43±0.14, ITA:0.36±0.08 | NI | NI | de Souza et al (2015b) |
|  | Various breeds | | | 1.5–6.9 years (4.3±0.3 years) | 31 | | | 33.7±0.6 kg | NI | NI | NI | 0.41 | 0.6 | NI | NI | England et al (2017) |
|  | Various breeds | | | 2–10 years | 22 | | | 11.6 ± 5.5 kg | NI | STA: 12.16 (21.9–8.3), MTA: 11.35 (19.6–7.0), ITA:5.7 (8.2–3.6) | STA: 5.6 (9.7–24), MTA:6.55 (11.0–3.4), ITA:3.47 (5.3–1.6) | STA: 0.55 (0.7–0.2), MTA:0.4 (0.6–0.1), ITA: 0.39 (0.5–0.1) | STA: 0.84 (1.5–0.2), MTA: 0.51 (1.0–0.2), ITA:0.48 (0.8–0.18) | NI | NI | Trautwein et al (2019) |
|  | Various breeds | | | 2–7 years old | 16 | | | 18–32 kg. | NI | STA: 19.7±2.8, MTA: 16.9±1.4, ITA:7±1.1 | STA: 8.3±0.4, MTA:7.8±0.8, ITA:4.5±0.2 | STA: 0.82±0.23, MTA:0.61±0.07, ITA:0.43±0.1 | STA: 0.51±0.06, MTA:0.44±0.04, ITA:0.32±0.06 | STA: 13.1±0.5, MTA:10.5±1.1, ITA:5.9±0.3 | NI | Gloria et al (2020) |
| **Species** | | **breeds** | **Age** | | | **No** | **B. W** | | **Season** | **Testicular hemodynamics in different portions of testicular arteries** | | | | | | **References** |
|  |  |  |  |  |  |  |  |  |  | **PSV** | **EDV** | **RI** | **PI** | **TAMAX** | **MV and Others** |  |
| **Dogs** | | Various breeds | 1.5-11.0 years (4.9±2.9 years) | | | 7 | 14.02±5.4 kg | | NI | STA: 11.79±2.78, MTA:23.19±5.92 | STA: 6.07±1.72, MTA: 9.29±1.96 | STA: 0.54±0.31, MTA: 0.61±0.82 | STA: 0.75±0.45, MTA:0.92±0.22, ITA: | NI | NI | Lemos et al (2020) |
|  |  | Various breeds | Various ages | | | 42 | Different BW | | NI | NI | NI | STA: 0.57, MTA:0.49, ITA: 0.47 | STA: 1.00, MTA: 0.78, ITA: 0.75 | NI | NI | Bigliardi et al (2019) |
|  |  | Various breeds | Various ages (<4 years to >7 years) | | | 30 | 9-17 kg | | NI | STA: 16.03±0.50, MTA:20.88±0.77 | STA: 7.85±0.38, MTA: 11.31±0.38 | STA: 0.50±0.01, MTA:0.45±0.02 | STA: 0.74±0.02, MTA:0.60±0.03 | STA: 11.28±0.33, MTA:15.47±0.47 | STA: SD=1.98±0.03, MTA: SD=1.79±0.05 | Günzel-Apel et al (2001) |
|  |  | Beagle dogs | 3-5 years | | | 5 | 16-23 kg | | NI | STA: 14.83±1.38 (11.09–18.56), MTA:10.81±1.38 (7.07–14.53), ITA:6.31±1.38 (2.57–10.03) | STA: 6.98±0.44 (5.86–8.11), MTA:6.33±0.44 (5.21–7.45), ITA: 4.09±0.44 (2.96–5.21) | STA: 0.52±0.19 (0.47–0.56), MTA: 0.41±0.19 (0.36–0.45), ITA: 0.34±0.19 (0.29–0.39) | STA: 0.83±0.19 (0.69–0.96), MTA:0.63±0.19 (0.51–0.76), ITA:0.49±0.19 (0.36–0.63) | NI | NI | Carrillo et al (2012) |
|  |  | Various breeds | 2-8 years | | | 20 | 10-42 Kg | | NI | STA: 13.77±0.80, MTA:11.97±0.84, ITA: 6.92±0.37 | STA: 6.25±0.53, MTA:7.32±0.54, ITA: 4.63±0.29 | STA: 0.54±0.04, MTA:0.39±0.03, ITA:0.34±0.02 | STA: 0.87±0.09, MTA:0.50±0.05, ITA:0.42±0.04 | NI | NI | Souza et al (2014) |
|  |  | Various breeds | 1-4 years | | | 20 | 8-12 kg | | NI | STA: 12.58±1.68, MTA:12.74±1.44 | STA: 5.56±1.07, MTA:7.64±1.04 | STA: 0.56±0.09, MTA:0.39±0.08 | STA: 0.94±0.26, MTA:0.53±0.19 | NI | NI | de Souza et al (2014) |

| **Species** | **breeds** | **Age** | **No** | **B. W** | **Season** | **Testicular hemodynamics in different portions of testicular arteries** | | | | | | **References** |
| --- | --- | --- | --- | --- | --- | --- | --- | --- | --- | --- | --- | --- |
|  |  |  |  |  |  | **PSV** | **EDV** | **RI** | **PI** | **TAMAX** | **MV and Others** |  |
| **Cats** | Shorthair cats | 3-5 years | 45 | 2.7-4.9 kg | NI | STA:  Left testis:  6.73 ± 2.78  Right testis:  6.23 ± 2.34 | STA:  Left testis:  2.80 ± 1.50  Right testis:  2.77 ± 1.36 | STA:  Left testis:  0.54 ± 0.12  Right testis:  0.53 ± 0.12 | NI | NI | NI | Brito (2015) |

**Abbreviations**

STA: Supratesticular artery

MTA: Marginal testicular artery

ITA: Intratesticular artery

PSV: Peak systolic velocity

EDV: End diastolic velocity

TAMAX: Time average maximum velocity

MV: Mean velocity

RI: Resistive index

PI: Pulsatility index

SD: Systole: diastole ratio

BFV: Blood flow volume

NI: Not identified in the literature

B. W: Body weight

No: Number
